# Supplementary material for: Topological magnons driven by the Dzyaloshinskii-Moriya interaction in the centrosymmetric ferromagnet Mn5Ge3
Source: Nat Commun. 2023 Nov 11;14:7321. doi: 10.1038/s41467-023-43042-3 (PMC10640582; doi:10.1038/s41467-023-43042-3)
Supplement: Supplementary file 1 — Supplementary Information [file 41467_2023_43042_MOESM1_ESM.pdf]

Supplementary Information for "Topological magnons driven by the Dzyaloshinskii-Moriya interaction in the centrosymmetric ferromagnet Mn_5Ge_3"

M. dos Santos Dias,[1,2,3,*] N. Biniskos,[4,†] F. J. dos Santos,[5,‡] K. Schmalzl,[6] J. Persson,[7] F. Bourdarot,[8] N. Marzari,[5,9] S. Blügel,[1] T. Brückel,[7] and S. Lounis[1,2]

[1]*Peter Grünberg Institut and Institute for Advanced Simulation, Forschungszentrum Jülich & JARA, D-52425 Jülich, Germany*

[2]*Faculty of Physics, University of Duisburg-Essen and CENIDE, D-47053 Duisburg, Germany*

[3]*Scientific Computing Department, STFC Daresbury Laboratory, Warrington WA4 4AD, United Kingdom*

[4]*Forschungszentrum Jülich GmbH, Jülich Centre for Neutron Science at MLZ, Lichtenbergstr. 1, D-85748 Garching, Germany*[§]

[5]*Theory and Simulation of Materials (THEOS), and National Centre for Computational Design and Discovery of Novel Materials (MARVEL), École Polytechnique Fédérale de Lausanne, 1015 Lausanne, Switzerland*

[6]*Forschungszentrum Jülich GmbH, Jülich Centre for Neutron Science at ILL, 71 Avenue des Martyrs, F-38000 Grenoble, France*

[7]*Forschungszentrum Jülich GmbH, Jülich Centre for Neutron Science (JCNS-2) and Peter Grünberg Institut (PGI-4), JARA-FIT, D-52425 Jülich, Germany*

[8]*Université Grenoble Alpes, CEA, IRIG, MEM, MDN, F-38000 Grenoble, France*

[9]*Laboratory for Materials Simulations, Paul Scherrer Institut, 5232 Villigen PSI, Switzerland*

(Dated: October 17, 2023)

CONTENTS

I. Supplementary experimental information 3
A. Synthesis of Mn_5Ge_3 3
B. Experimental setup 3
C. Inelastic neutron scattering measurements and data analysis 5
D. Magnon gap around the K-point 11

II. Supplementary theoretical information 12
A. Structural optimization 12
B. First-principles calculations of the magnetic interactions 13
C. Theoretical framework for the calculation of the magnon spectrum 16
1. Semiclassical spin dynamics 17
2. Eigenvalues and eigenvectors of the dynamical matrix 19
3. Dynamical magnetic susceptibility 20
4. Dynamical structure factor 20
D. Theoretical dependence of the magnon bands on the structural parameters 22
E. Simplified effective spin model 23
F. Simulated INS using the effective spin model 27

Supplementary References 30

* m.dos.santos.dias@fz-juelich.de
† nikolaos.biniskos@matfyz.cuni.cz
‡ flaviano.dossantos@psi.ch
§ Current address: Charles University, Faculty of Mathematics and Physics, Department of Condensed Matter Physics, Ke Karlovu 5, 121 16, Praha, Czech Republic

I. SUPPLEMENTARY EXPERIMENTAL INFORMATION

A. Synthesis of Mn_5Ge_3

Elementary manganese (purity 99.99 %) and germanium (purity 99.9999 %) were used for synthesizing polycrystalline samples of Mn_5Ge_3. The elements were mixed in stoichiometric ratios and melted in argon atmosphere by induction heating in a levitation cold crucible. The resulting product was cooled and heated four times to ensure maximum homogeneity of the sample. In order to confirm the formation of the Mn_5Ge_3 phase, part of the polycrystalline sample was characterized by X-ray powder diffraction at room temperature. When the formation of Mn_5Ge_3 was confirmed, a single crystal was grown from the polycrystalline samples by the Czochralski technique. The powder was inserted in an aluminum oxide crucible and a tungsten crystal was used as seed. Once the crystal was grown, a small part of it was ground and X-ray powder diffraction at room temperature confirmed its phase purity. The Mn_5Ge_3 crystal was oriented with a Laue camera and was cut by spark erosion perpendicular to the hexagonal [100] and [001] crystallographic directions. Further details regarding the characterisation of samples of this batch can be found in Ref. [1].

B. Experimental setup

The single crystal used for inelastic neutron scattering (INS) experiments (diameter $\approx$ 8 mm, height $\approx$ 20 mm, mass $\approx$ 10.2 g) was mounted on an aluminium sample holder (see Fig. S1). The linewidths of the rocking curves of the sample consist of single Gaussian peaks of about 0.48° as determined by neutron measurements performed on the triple axis spectrometers (TASs) at the Institut Laue-Langevin (ILL) (see Figs. S2). The lattice parameters of Mn_5Ge_3 obtained at $T = 10\,\mathrm{K}$ are $a = b = 7.059$ Å and $c = 4.949$ Å. To obtain the Curie temperature of Mn_5Ge_3 we measured the temperature dependence at $\mathbf{Q} = (1.95, 0, 0)$ and $E = 0.5\,\mathrm{meV}$ with configuration "A" (see Table SI). The obtained intensity after background subtraction was corrected by the detailed balance factor so that the final result relates to the imaginary part of the dynamical spin susceptibility (see Fig. S1). The maximum of $\chi"(\mathbf{Q}, E)$ is observed at $T_C \approx 292\,\mathrm{K}$.

Unpolarized INS measurements were carried out on the CRG-Jülich and CRG-CEA Grenoble cold and thermal TASs IN12 [2] and IN22, respectively. The spectrometers used for

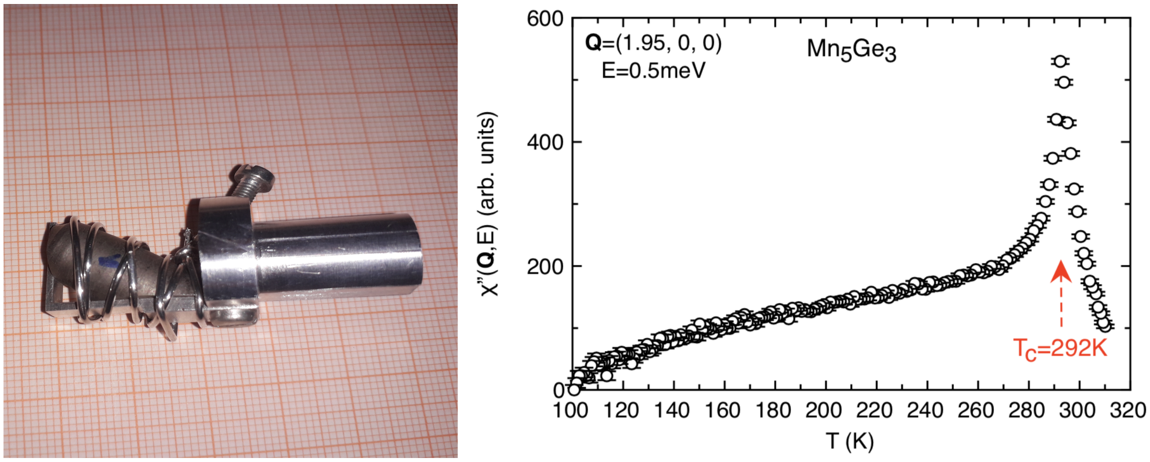

Fig. S1. **Mn_5Ge_3 single crystal and its ordering temperature. Left:** Mn_5Ge_3 single crystal used for neutron scattering experiments. Aluminum wire was added to ensure the stability of the sample on the aluminum sample holder during the measurements under external magnetic field. **Right:** Temperature dependence of the dynamical spin susceptibility $\chi"(\mathbf{Q}, E)$ at $\mathbf{Q} = (1.95, 0, 0)$ and $E = 0.5\,\mathrm{meV}$. The red arrow indicates the maximum of $\chi"(\mathbf{Q}, E)$ at $T_C \approx 292\,\mathrm{K}$. The error bars indicate one standard deviation (square root of the neutron counts).

INS studies were setup in W configuration with a fixed final energy. Bent pyrolytic graphite crystals were used as the monochromator and the analyzer, respectively, while open collimations were installed to fully benefit from the focusing effects. Additional information regarding each configuration is given in Table SI.

To cover the temperature region of $10 \leq T \leq 316\,\mathrm{K}$ the sample was placed inside a ^{4}He

Configuration	TAS	Monochromator	Analyzer	Filter	$\lvert\mathbf{k}_f\rvert$ (Å^{-1})	Sample Orientation
A	IN22	PG(002)	PG(002)	PG	2.662	[100]/[010] and [100]/[001]
B	IN12	PG(002)	PG(002)	PG	1.971	[100]/[010]
C	IN12	PG(002)	PG(002)	Be, PG	1.55, 1.971	[100]/[010]

Table SI. **Instrument configurations.** "PG" refers to pyrolytic graphite. Higher order contamination was removed using a PG or a beryllium (Be) filter in the scattered neutron beam. Inelastic scans were performed with constant $\lvert\mathbf{k}_f\rvert$, where $\mathbf{k}_f$ is the wave-vector of the scattered neutron beam.

flow cryostat and we used configurations "A" and "B" to investigate the spin excitation spectrum of Mn_5Ge_3. For the temperature range of $10 \leq T < 500$ K we used a cryofurnace. To access different high symmetry directions the single crystal was oriented either in the [100]/[010] or in the [100]/[001] scattering planes of the hexagonal lattice.

Spin dynamics investigations under magnetic field were carried out using a 3.5 T horizontal field magnet, a 2.5 T vertical field magnet and configuration "C" (see Table SI). The crystal was oriented in the [100]/[010] scattering plane of the hexagonal symmetry, was first heated up at the maximum available temperature and then cooled down to 10 K under the maximum available field applied parallel to the desired direction (a^* or c). This resulted in a single domain state of the sample. Although the magnetic moments point along the hexagonal c axis, Mn_5Ge_3 is a "soft" ferromagnet and the magnetization approaches saturation for a field of about 1.5 T applied perpendicular to the easy axis at 280 K [1].

C. Inelastic neutron scattering measurements and data analysis

The magnons of Mn_5Ge_3 were investigated as a function of the wave-vector and the energy transfer E. In this work we use the hexagonal coordinate system and the scattering vector $\mathbf{Q} = (Q_h, Q_k, Q_l)$ is given in reciprocal lattice units (r.l.u.). The wave-vector $\mathbf{q}$ is related to the momentum transfer through $\hbar\mathbf{Q} = \hbar\mathbf{G} + \hbar\mathbf{q}$, where $\mathbf{G}$ is a Brillouin zone center and $\mathbf{G} = (h, k, l)$. For all neutron raw data shown in the main text and the supplementary material, the error bars are taken as the square root of the neutron counts, which corresponds to one standard deviation.

Magnetic excitations were measured around different Brillouin zone centers for energy transfers $0 \leq E \leq 40$ meV. Intense spin-wave scattering was observed around the zone centers $\mathbf{G} = (2, 0, 0)$ and $\mathbf{G} = (0, 0, 2)$ where the magnetic form factor for Mn is expected to have significant magnitude [3]. Scans at constant E and constant $\mathbf{Q}$-scans were carried out at $T = 10$ K along the hexagonal high-symmetry directions $\Gamma - \mathrm{M}$, $\Gamma - \mathrm{K} - \mathrm{M}$, and $\Gamma - \mathrm{A}$ (see Figs. S3, S4, S5, S6, S7, S8, S9). Specific scans were repeated above T_C, in order to establish the magnetic nature of the excitations.

Since the resolution ellipsoid of a TAS is usually more elongated along one direction, we performed most measurements where the focusing condition of the spectrometer is fulfilled. For the focusing condition, the long axis of the ellipsoid is parallel to the dispersion curve,

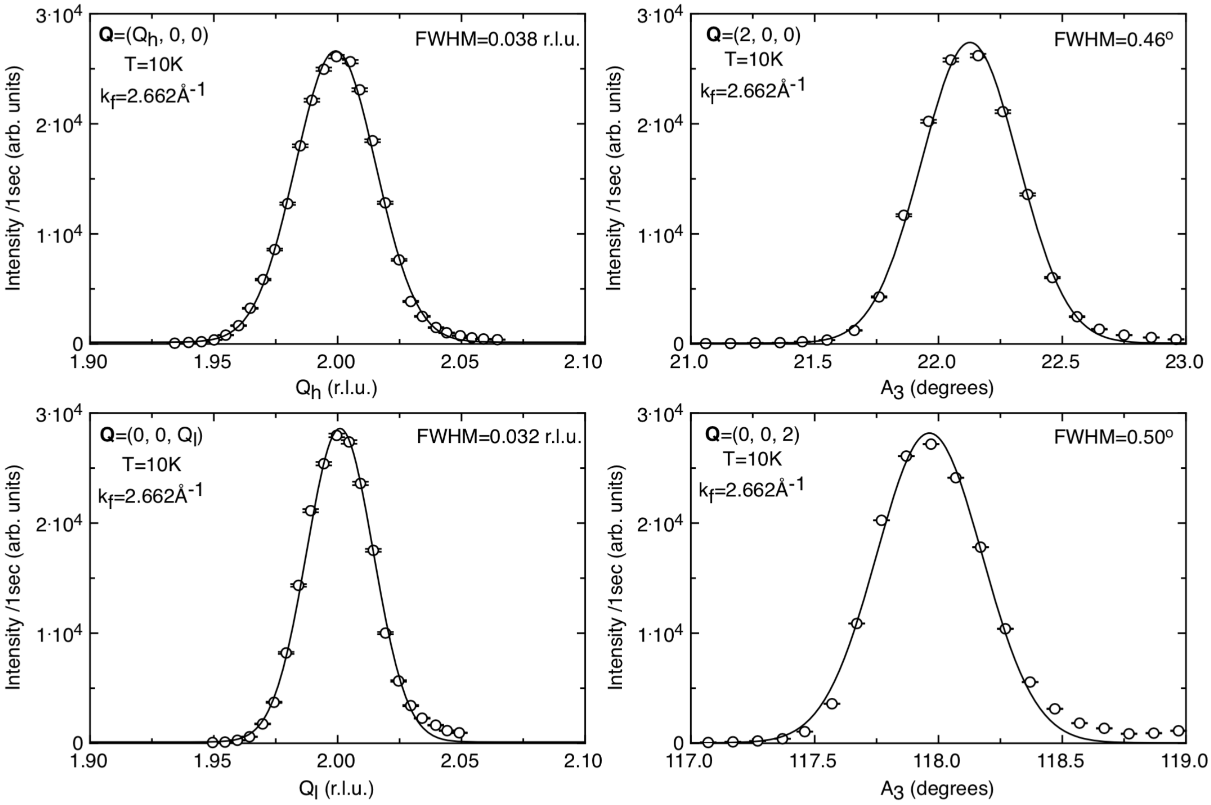

Fig. S2. **Alignment scans for the Mn_5Ge_3 single crystal. Left:** Alignment scans ($E = 0\,\text{meV}$) of the Mn_5Ge_3 crystal around the Bragg peaks $\mathbf{Q} = (2, 0, 0)$ and $\mathbf{Q} = (0, 0, 2)$ at $T = 10\,\text{K}$ with configuration "A" (see Table SI). **Right:** The mosaic was estimated from the full width at half maximum (FWHM) of the rocking curves of the sample. Solid lines indicate Gaussian fits. To avoid damaging and saturating the detector a 10 mm Plexiglas attenuator was in place. The error bars indicate one standard deviation (square root of the neutron counts).

therefore, the observed peaks will be more intense and narrower compared to the ones obtained with the defocusing condition. Typical representatives of such measurements are shown in Figs. S3.

To analyze the obtained spectra at $T = 10\,\text{K}$ a constant background was assumed for the $\mathbf{Q}$-scans, while for the E-scans a linear background was used. Gaussian functions were selected to describe the peaks (e.g. see Fig. S4(a)). For the data obtained with constant $\mathbf{Q}$-scans the signal centered at $E = 0\,\text{meV}$ corresponds to the elastic line. The peaks observed at finite E at $T = 10\,\text{K}$ are replaced by a broad quasi-elastic signal above the ordering temperature at $T = 316\,\text{K} \approx 1.082 \times T_C$ and at $T = 398\,\text{K} \approx 1.36 \times T_C$ (e.g. see

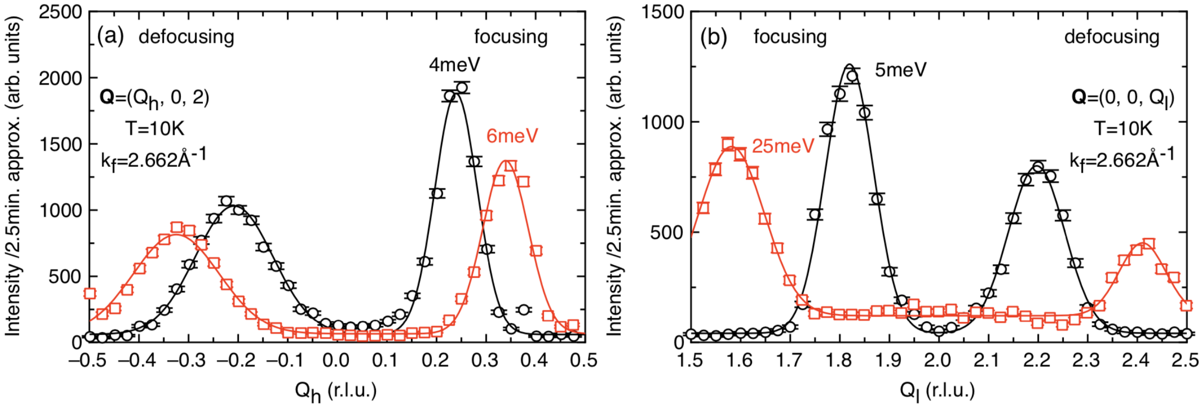

Fig. S3. **Focusing and defocusing condition for INS spectra.** The measurements were performed at 10 K at different constant energy transfers around **a**: $\mathbf{Q} = (Q_h, 0, 2)$ and **b**: $\mathbf{Q} = (0, 0, Q_l)$. The error bars indicate one standard deviation (square root of the neutron counts).

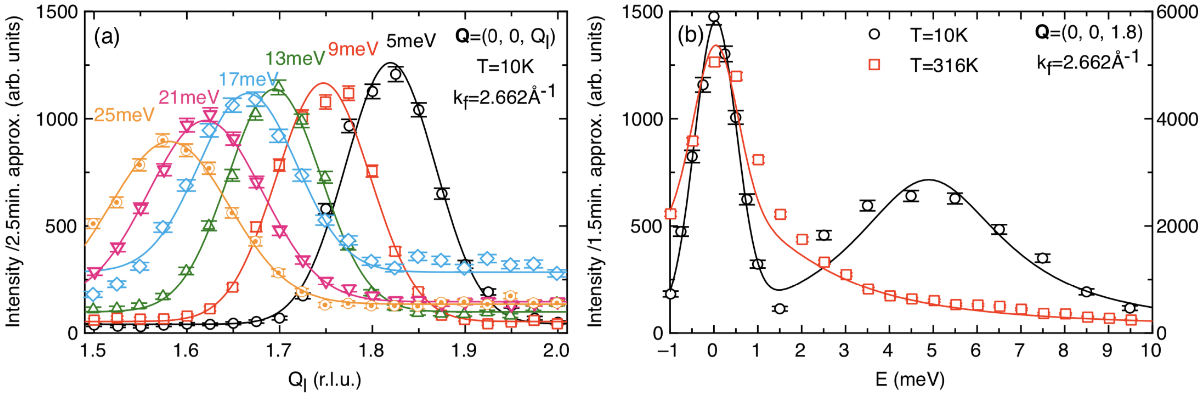

Fig. S4. **Inelastic spectra for obtaining magnons along the $\Gamma - \mathbf{A}$ direction.** **a** **Q**-scans around $\mathbf{Q} = (0, 0, Q_l)$ at different constant energy transfers at 10 K. **b** Energy spectra at $\mathbf{Q} = (0, 0, 1.8)$ measured at 10 K (black circles) and 316 K $\approx 1.082 \times T_C$ (red squares). Neutron intensity for the data at 10 K and 316 K is given on the left and right vertical axis, respectively. Note: $\mathbf{q} \parallel \mathbf{Q}$. The error bars indicate one standard deviation (square root of the neutron counts).

Figs. S4(b), S5(b), S6(d), S7(b), S8(b)) confirming the magnetic nature of the excitations. A Lorentzian function was used to describe the signal in the paramagnetic phase. The “holes” in the spectra (discontinued data points) correspond to spurious scattering that has been masked during the data evaluation. For determining the spin gap at the K-point at zero field we collected energy spectra at different Brillouin zones at 10 K (see Fig. S9(b)).

We observed that the second spin-wave mode along $\Gamma - \mathrm{K}$ for $q < 1.5$ r.l.u. and $E <$

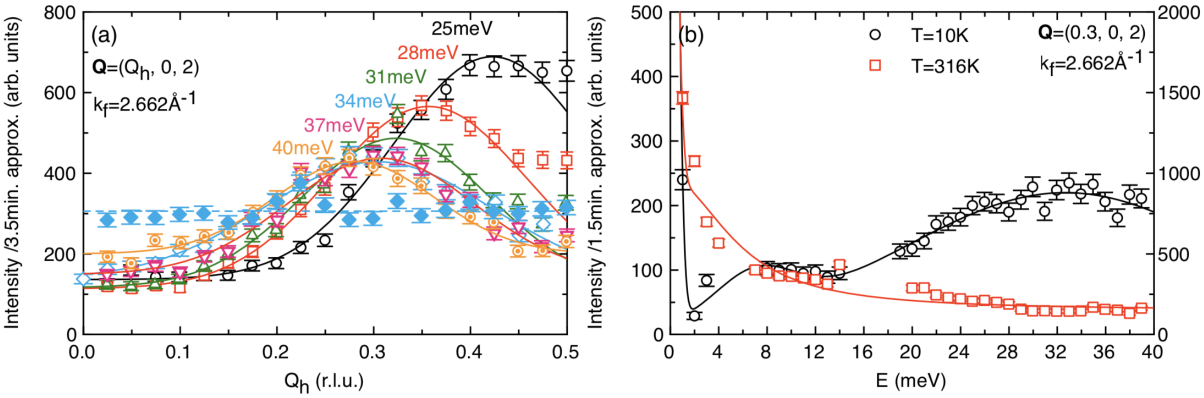

Fig. S5. **INS measurements for obtaining the optic magnon branch along the $\Gamma - \mathrm{M}$ direction. a Q**-scans around $\mathbf{Q} = (Q_h, 0, 2)$ at different constant energy transfers at 10 K (empty symbols). Full blue symbols correspond to data measured at 316 K $\approx 1.082 \times T_C$ at $E = 34$ meV. The dashed blue line is a guide for the eyes. **b** Energy spectra at $\mathbf{Q} = (0.3, 0, 2)$ measured at 10 K (black circles) and 316 K (red squares). Neutron intensity for the data at 10 K and 316 K is given on the left and right vertical axis, respectively. Note: $\mathbf{q} \perp \mathbf{Q}$. The error bars indicate one standard deviation (square root of the neutron counts).

10 meV (see Fig. 2b in the main text) is better described by a linear-like dispersion relation that resembles AFM spin-waves or lattice excitations. However, studies in bulk Mn_5Ge_3 indicate that it is characterized by strong FM correlations with no clear evidence of any latent AFM, therefore, this unexpected behavior could possibly originate from two mechanisms. A strong coupling between charge and bosonic degrees of freedom can result in hybridized collective modes. Neutron spectroscopic studies can identify the magnetic and phononic component of such excitations, which are referred as magnon-polarons (magneto-elastic modes), and are reported in several magnetic materials, e.g. $YMnO_3$ [4] and Mn_3Ge [5]. In the case of Mn_5Ge_3 a coupling of charge carriers to bosonic modes has been identified by infrared spectroscopy on single crystals and their interaction energy was estimated to be about 60 meV [6]. Another study using angle-resolved photoemission spectroscopy on Mn_5Ge_3(001) films epitaxially grown on Ge(111) proposed the formation of electron-polaron modes for the explanation of the spectral weight at the Fermi level [7]. Building up on these results, the calculated phonon dispersion relations of Mn_5Ge_3 [8] indicate that longitudinal acoustic phonons are in the proximity of the linear-like magnetic excitations observed with INS. Therefore, these modes may relate to magnon-polarons, resulting in a deviation of the

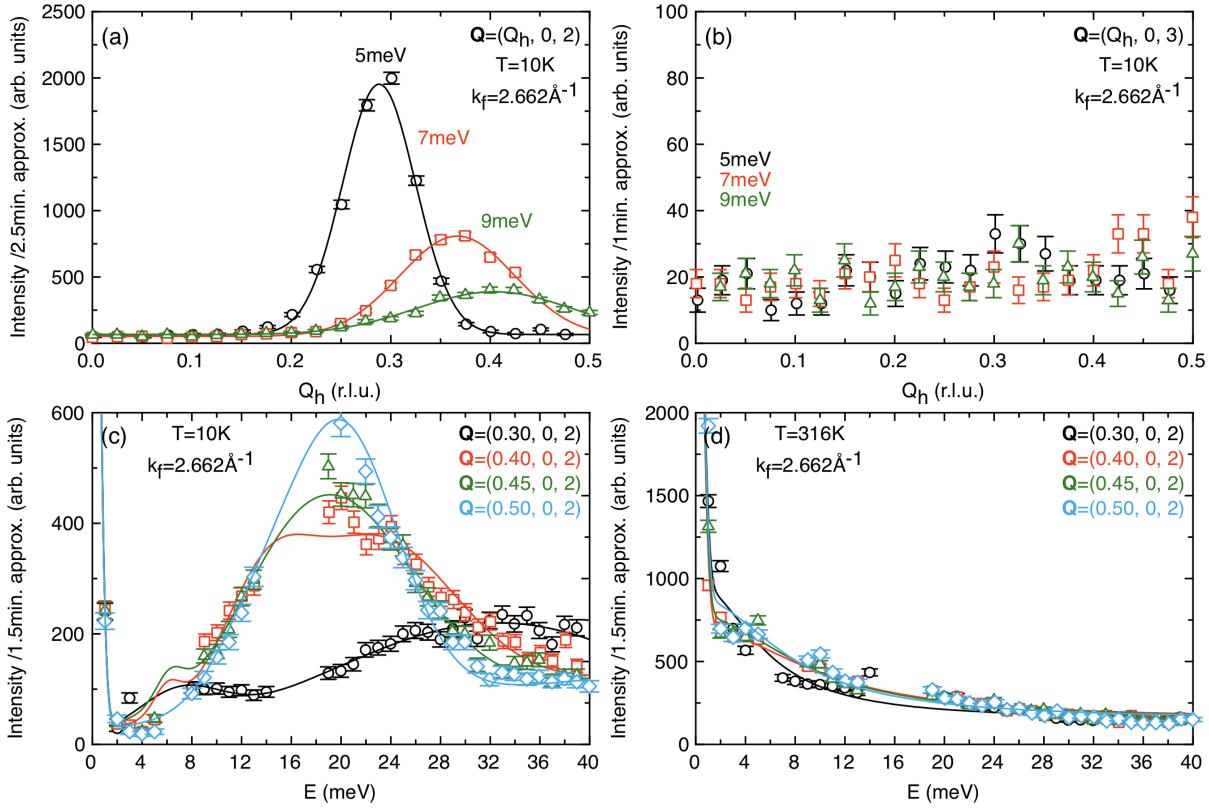

Fig. S6. **INS measurements for obtaining the magnon modes along the $\Gamma - \mathrm{M}$ direction.** **Q**-scans at different constant low energy transfers at 10 K around **a**: $\mathbf{Q} = (Q_h, 0, 2)$ and **b**: $\mathbf{Q} = (Q_h, 0, 3)$. Peaks are not observed around the zone center $\mathbf{G} = (0, 0, 3)$. Energy spectra at different Q_h positions measured around $\mathbf{G} = (0, 0, 2)$ at **c**: 10 K and **d**: at 316 K $\approx 1.082 \times T_C$. Note: $\mathbf{q} \perp \mathbf{Q}$. The error bars indicate one standard deviation (square root of the neutron counts).

expected quadratic behavior for FM spin-waves close to the Γ-point. Another scenario for this unusual behaviour may have its origin in a part of the neutron scattering cross section for magnetic scattering, namely the magnetovibrational (MV) scattering term, which is the creation or annihilation of a phonon via the magnetic interaction [9, 10]. MV scattering is often associated with parasitic scattering [11], however, in some cases it has been used to study magnetic form factors [12] or spin-lattice coupling [13]. Although in our study we measured excitations in the lowest accessible Brillouin zones, it is possible to obtain phonon-like modes through MV scattering even in the first Brillouin zone, where **Q** has the smallest magnitude. In addition, the temperature dependence of such modes will be similar to magnetic scattering. Finally, we note that we obtained the peak positions of the

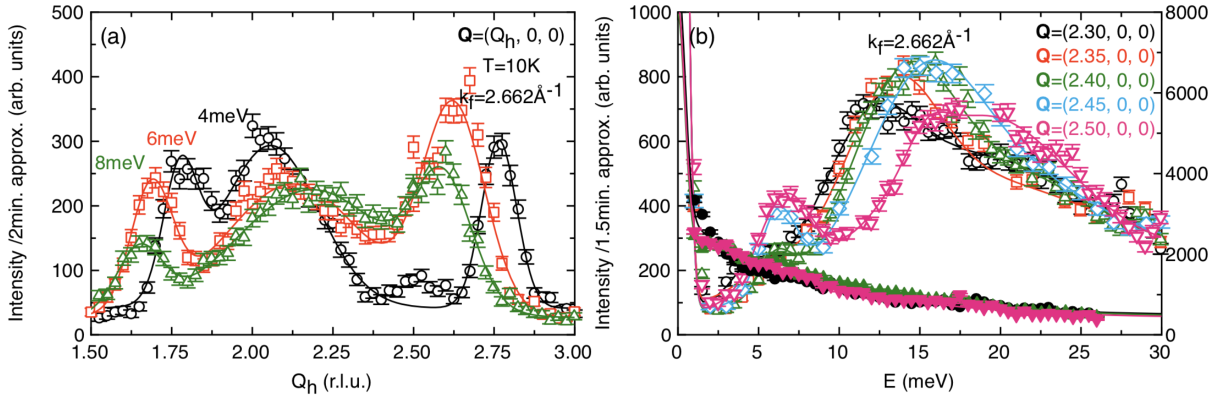

Fig. S7. **INS measurements for obtaining the magnon modes along the $\Gamma - \mathrm{M}$ direction.** **a** **Q**-scans at different constant low energy transfers at 10 K. **b** Energy spectra at different Q_h positions measured around $\mathbf{G} = (2, 0, 0)$ at 10 K (empty symbols) and at 316 K $\approx 1.082 \times T_C$ (full symbols). Neutron intensity for the data at 10 K and 316 K is given on the left and right vertical axis, respectively. Note: $\mathbf{q} \parallel \mathbf{Q}$. The error bars indicate one standard deviation (square root of the neutron counts).

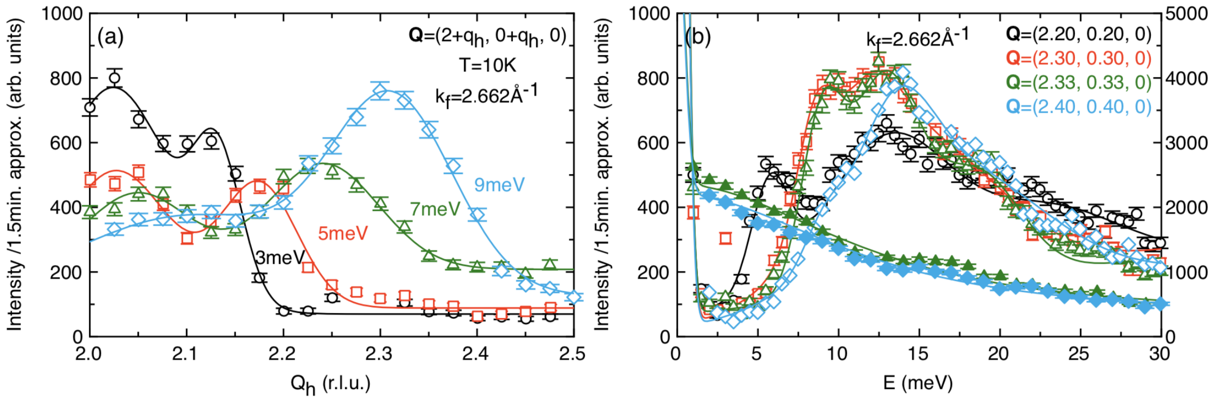

Fig. S8. **INS measurements for obtaining the magnon modes along the $\Gamma - \mathbf{K} - \mathrm{M}$ direction.** **a** **Q**-scans at different constant low energy transfers at 10 K. **b** Energy spectra at different Q_h positions measured around $\mathbf{G} = (2, 0, 0)$ at 10 K (empty symbols) and at 398 K $\approx 1.36 \times T_C$ (full symbols). Neutron intensity for the data at 10 K and 398 K is given on the left and right vertical axis, respectively. Note: $\mathbf{q} \parallel \mathbf{Q}$. The error bars indicate one standard deviation (square root of the neutron counts).

second magnon modes close to the Γ-point ($q < 1.5$ r.l.u.) with scans at constant-E and only in scattering geometries where $\mathbf{q} \parallel \mathbf{Q}$ (see Fig. S8(a)). In contrast, all other excitations

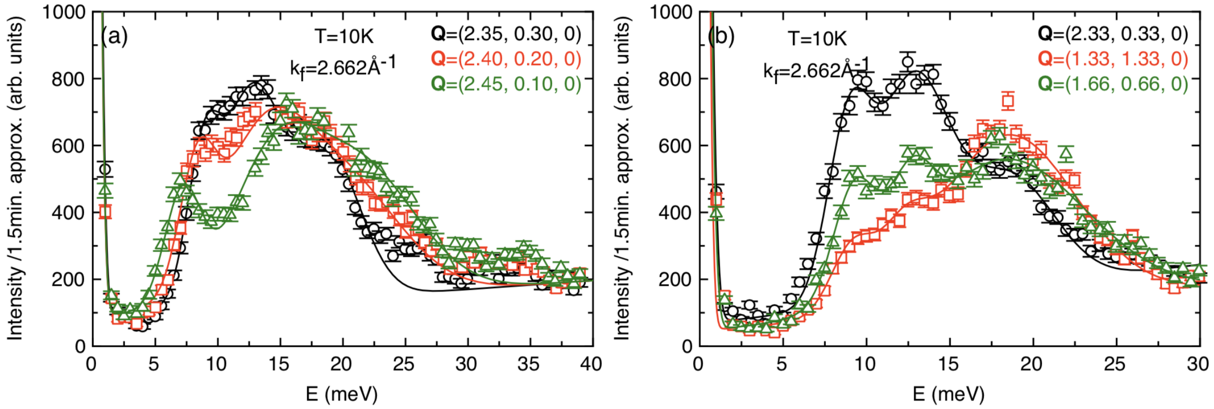

Fig. S9. **INS measurements to investigate magnon modes relating to the K-point. a** Energy spectra for obtaining the magnon modes along the $\Gamma - \mathrm{K} - \mathrm{M}$ direction at different Q_h positions measured around $\mathbf{G} = (2,0,0)$ at 10 K (note: $\mathbf{q} \perp \mathbf{Q}$). **b** Energy spectra at different K-points obtained at 10 K. The error bars indicate one standard deviation (square root of the neutron counts).

along the $\Gamma - \mathrm{K} - \mathrm{M}$ path are observed both in geometries where $\mathbf{q} \parallel \mathbf{Q}$ and $\mathbf{q} \perp \mathbf{Q}$ (see Figs. S8(b), S9). We point out that the results of our Letter are not affected from the origin of this linear-like behaviour close to the Γ-point for the second magnon mode.

D. Magnon gap around the K-point

In Figs. S10 we show INS data around the K-point obtained along the Γ–K and K–M paths of the hexagonal symmetry. In zero magnetic field when the magnetization is along the c-axis two peaks are visible. The application of a magnetic field in the ab-plane merges the two peaks into one, demonstrating the closure of the magnon energy gap at different $\mathbf{Q}$ positions close to the K-point. The integrated intensity of the single peak obtained for a magnetic field applied along the a^*-axis almost equals to the sum of the two individual peaks observed at zero field (when the magnetization is $\parallel \mathbf{c}$). We note that around the K-point when the magnetization is along the a^*-axis, the dispersing Dirac modes are too close in energy and cannot be separately resolved within our instrumental resolution. The observed behaviour is consistent with the one shown in Fig. 3b of the main text for the K-point.

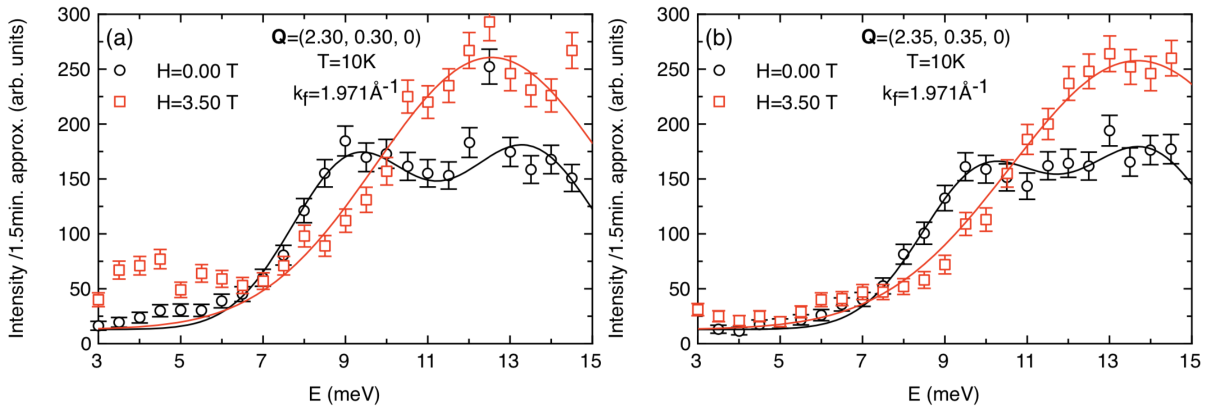

Fig. S10. **Field dependence (H $\parallel$ a*) of the magnon modes around the K-point. a** Energy spectra at $\mathbf{Q} = (2.30, 0.30, 0)$ (Γ–K path of hexagonal symmetry). **b** Energy spectra at $\mathbf{Q} = (2.35, 0.35, 0)$ (K–M path of hexagonal symmetry). Measurements performed at 10 K. The error bars indicate one standard deviation (square root of the neutron counts).

II. SUPPLEMENTARY THEORETICAL INFORMATION

A. Structural optimization

The optimization of the crystal structure parameters was carried out with the Quantum Espresso package [14]. Ultrasoft pseudopotentials from the GBRV library v1.5 [15] were used with the Perdew-Burke-Ernzerhof functional for exchange and correlation [16]. The plane-wave cutoffs for the wave functions and the density were set to 70 Ry and 840 Ry, respectively. The Brillouin zone summations were performed with a Monkhorst-Pack grid of $12\times12\times18$ and Gaussian smearing with a smearing parameter of 3 mRy. The self-consistency cycle was deemed converged when the change in the total energy falls below 10^{-9} Ry. The system was initialized in the ferromagnetic state and the internal coordinates and the unit cell dimensions were allowed to change, starting from the experimental structure given in Ref. [17], until the change in the total energy and in the forces were below 10^{-4} in their respective atomic Rydberg units.

The results of the structural relaxation are given in Table SII together with experimental reference values. Comparing with Ref. [17] we find overall very good agreement, with $\Delta a = -0.7\,\%$, $\Delta c = -1.6\,\%$, $\Delta x = 1.5\,\%$ for Mn_2 and $\Delta x = 0.5\,\%$ for Ge.

	a (Å)	c (Å)	c/a	x for Mn_2	x for Ge
Ref. [17]	7.184	5.053	0.7034	0.2397	0.6030
QE	7.1358	4.9704	0.6919	0.2434	0.6058

Table SII. **Crystal structure parameters for Mn_5Ge_3.** Mn_1 occupies the $4d$ site with coordinates $(1/3, 2/3, 0)$ and Mn_2 and Ge the $6g$ sites with coordinates $(x, 0, 1/4)$. The parameters for the relaxed structure obtained with Quantum Espresso (QE) are compared to the experimental values.

B. First-principles calculations of the magnetic interactions

In order to address the magnetism of Mn_5Ge_3, we employ the all-electron Korringa-Kohn-Rostoker Green's function method using full potential [18] with spin-orbit coupling added to the scalar relativistic approximation [19] as implemented in the juKKR code package [20]. The exchange-correlation functional is the local spin-density approximation as parameterized by Vosko, Wilk and Nusair [21]. The scattering wave functions are expanded up to an angular momentum cutoff of $\ell_{\max} = 3$, the energy integrations are carried out in the upper complex energy plane with a Fermi-Dirac smearing of $T = 502.78\,\mathrm{K}$ [22], and the k-point summations use a grid of $24 \times 24 \times 34$ points.

Via the relativistic extension of the infinitesimal rotation approach [23], the juKKR code package gives access to the tensor of pairwise magnetic exchange interactions featuring in the following spin hamiltonian:

$$\begin{aligned}\mathcal{H} &= -\sum_{n}\sum_{\mu,\nu}\sum_{\alpha,\beta} J^{\alpha\beta}_{0\mu,n\nu} S^{\alpha}_{0\mu} S^{\beta}_{n\nu} \\ &= -\sum_{n}\sum_{\mu,\nu}\left(J_{0\mu,n\nu}\mathbf{S}_{0\mu}\cdot\mathbf{S}_{n\nu} + \mathbf{D}_{0\mu,n\nu}\cdot(\mathbf{S}_{0\mu}\times\mathbf{S}_{n\nu}) + \sum_{\alpha,\beta}\Delta J^{\alpha\beta}_{0\mu,n\nu} S^{\alpha}_{0\mu} S^{\beta}_{n\nu}\right) . \qquad \text{(S1)}\end{aligned}$$

The location of the magnetic atoms is specified by a unit cell index n (with 0 the reference unit cell) and the different atoms within the unit cell are labelled with μ and ν. The spin components with α and β and the spin length scaled to unity, $|\mathbf{S}| = 1$. The magnetic exchange interactions comprise the spin-isotropic Heisenberg exchange $J_{0\mu,n\nu}$, the Dzyaloshinskii-Moriya interaction $\mathbf{D}_{0\mu,n\nu}$, and the two-site anisotropy $\Delta J^{\alpha\beta}_{0\mu,n\nu}$. Both $\mathbf{D}_{0\mu,n\nu}$ and $\Delta J^{\alpha\beta}_{0\mu,n\nu}$ arise from the spin-orbit interaction. In addition, $\mathbf{D}_{0\mu,n\nu}$ vanishes if there is an

inversion center in the middle of the bond connecting a given pair of magnetic atoms, which is one of the famous symmetry rules identified by Moriya [24]. The single-site anisotropy can be formally included as the $n = 0$, $\mu = \nu$ (site-diagonal) part of the $\Delta J^{\alpha\beta}_{0\mu,n\nu}$ tensor.

The spin magnetic moments for Mn1 and Mn2 are $2.11\,\mu_\mathrm{B}$ and $3.14\,\mu_\mathrm{B}$ for the experimental crystal structure and $2.01\,\mu_\mathrm{B}$ and $3.04\,\mu_\mathrm{B}$ for the theoretically optimized structure. We first discuss the effective magnetic anisotropy energy. Mn1 is in the Wyckoff position $4d$ which has 3.2 symmetry, so its effective anisotropy is of the uniaxial type. Writing $\mathcal{H} = K_z S_z^2$, our DFT calculations give $K_z \approx 0.07$ meV for the Mn1 sites. Mn2 is in the Wyckoff position $6g$ which has $m2m$ symmetry and is described by a biaxial anisotropy. Defining a local set of axes so that z is the c-axis and y is the normal to the mirror plane passing by the atom, we write $\mathcal{H} = K_{xy}\left(S_x^2 - S_y^2\right) + K_z S_z^2$ and our calculations resulted in $K_{xy} \approx -0.12$ meV and $K_z \approx -0.18$ meV, respectively. Adding up all the contributions for a ferromagnetic state, the K_{xy} contributions from the different Mn2 sites cancel out, and we have $K_z^{\mathrm{total}} = 4 \times 0.07 + 6 \times (-0.18) = -0.80$ meV, which rounds up to -0.1 meV per Mn atom, as quoted in the main text.

Next we consider the intersite interactions. Table I of the main text shows a comparison between our computed magnetic interactions using the experimental crystal structure with the values reported in Ref. [25], and we find good quantitative agreement. The variation of the magnetic interactions with the distance between Mn atoms is shown in Fig. S11. We find that the interactions are long-ranged and many more have to be taken into account beyond the tabled J_1–J_6 ones in order to have a converged magnon dispersion. We also compare our values obtained using the experimental crystal structure with those obtained using the theoretically optimized one. We see that the bond lengths differ very slightly between the two structures. The FM interactions are large and so change little in relative terms, while the AFM interactions being small show a larger relative change and weaken when going from the experimental to the theoretical structure. This weakening of the AFM interactions is responsible for the large change in the theoretical magnon spectrum shown in Fig. 1c of the main text.

The Hamiltonian presented in Eq. 1 of the main text is the minimal one that explains the magnon properties of Mn_5Ge_3, while the one given in Eq. (S1) is the one that represents all the magnetic interactions that are obtained from our DFT calculations. Starting from the more complex Hamiltonian of Eq. (S1) and analysing the obtained values of the mag-

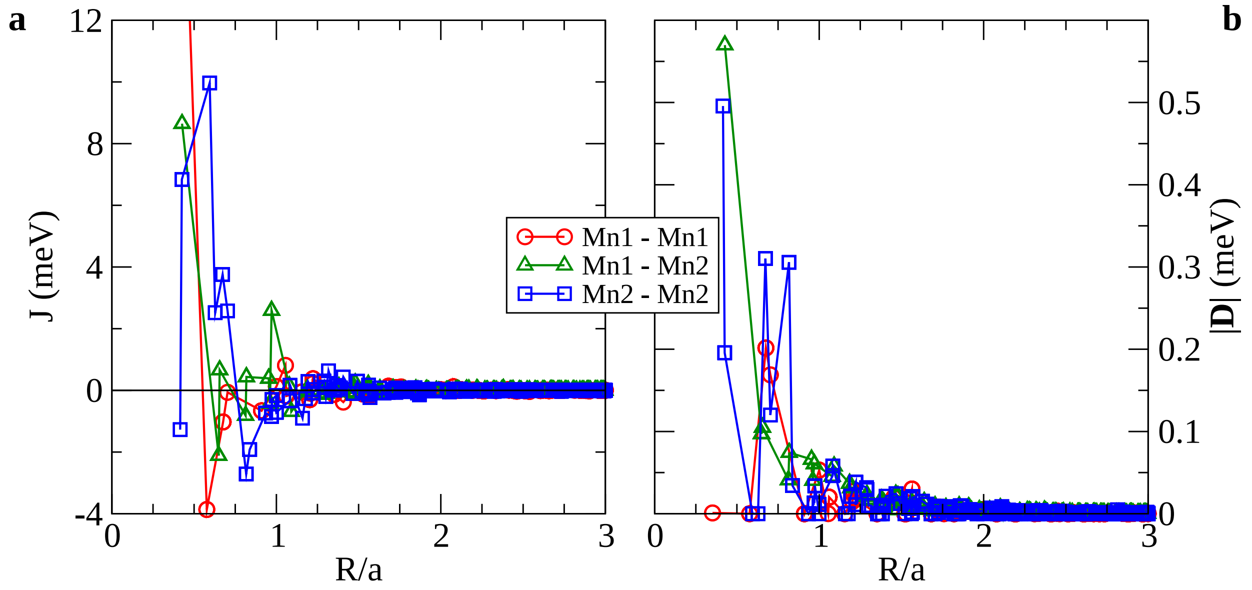

Fig. S11. **Magnetic exchange interactions computed from DFT as a function of the distance between pairs of Mn atoms. a** Heisenberg exchange interaction. The first Mn1-Mn1 interaction is not visible due to its large magnitude. **b** Magnitude of the Dzyaloshinskii-Moriya interaction. The experimental crystal structure was considered. The distance between Mn atom pairs R is given in units of the lattice parameter a.

netic interactions and the corresponding magnon bands, we concluded that the simplified Hamiltonian in Eq. 1 of the main text leads to almost indistinguishable results, and so we decided to use it to explain our results. This is due to a natural hierarchy of the magnetic interactions in Mn_5Ge_3, a material that does not contain heavy elements. As the spin-orbit coupling (SOC) is rather weak, we have that the Heisenberg exchange interactions are the strongest (zeroth-order in SOC), the DMI are the second-strongest (first-order in SOC; only allowed if the pair of Mn atoms is not related by inversion), and the symmetric anisotropic interactions (including the single-ion anisotropy) are the weakest (second-order in SOC). The overall weakness of the symmetric anisotropic interactions is corroborated by the experimental value of the uniaxial magnetic anisotropy and by the expected magnon gap at Γ not being resolvable with the employed INS setup.

C. Theoretical framework for the calculation of the magnon spectrum

It can be shown that linearized spin wave theory [26] agrees with the results of the linearized semiclassical Landau-Lifshitz equation. As the latter is a more intuitive approach to obtain the dynamical spin susceptibility which is needed for comparison with inelastic neutron scattering, we sketch the corresponding derivation in the following.

Consider the following generalized Heisenberg model:

$$\mathcal{H} = -\sum_i \sum_\mu \sum_\alpha M_\mu B^\alpha_{i\mu}\, S^\alpha_{i\mu} - \sum_i \sum_\mu \sum_{\alpha,\beta} S^\alpha_{i\mu}\, K^{\alpha\beta}_\mu\, S^\beta_{i\mu} - \frac{1}{2}\sum_{i,j}\sum_{\mu,\nu}\sum_{\alpha,\beta} S^\alpha_{i\mu}\, J^{\alpha\beta}_{i\mu,j\nu}\, S^\beta_{j\nu}\ . \quad \text{(S2)}$$

The spins are classical and assumed to have unit length ($|\mathbf{S}_i| = 1$; the magnitude of the spin moments is M_μ), and are assigned to atomic sites $\mathbf{R}_{i\mu} = \mathbf{R}_i + \mathbf{R}_\mu$, with $\mathbf{R}_i$ the N vectors of the Bravais lattice and $\mathbf{R}_\mu$ the basis vectors inside the unit cell. Here $B^\alpha_{i\mu}$ is a generalized magnetic field for later purposes. The onsite anisotropy matrix can be written in general as

$$K_\mu = \begin{pmatrix} K^{xx}_\mu & K^{xy}_\mu & K^{xz}_\mu \\ K^{xy}_\mu & K^{yy}_\mu & K^{yz}_\mu \\ K^{xz}_\mu & K^{yz}_\mu & K^{zz}_\mu \end{pmatrix}\ . \quad \text{(S3)}$$

The magnetic exchange interactions can be split into Heisenberg, Dzyaloshinskii-Moriya and two-site anisotropy contributions,

$$\sum_{\alpha,\beta} S^\alpha_{i\mu}\, J^{\alpha\beta}_{i\mu,j\nu}\, S^\beta_{j\nu} = J_{i\mu,j\nu}\, \mathbf{S}_{i\mu}\cdot\mathbf{S}_{j\nu} + \mathbf{D}_{i\mu,j\nu}\cdot(\mathbf{S}_{i\mu}\times\mathbf{S}_{j\nu}) + \sum_{\alpha,\beta} S^\alpha_{i\mu}\, \Delta J^{\alpha\beta}_{i\mu,j\nu}\, S^\beta_{j\nu}\ . \quad \text{(S4)}$$

Due to the translational invariance of the Bravais lattice, the spins can also be represented by the components of their lattice Fourier transform,

$$\mathbf{S}_{i\mu} = \sum_{\mathbf{k}} e^{\mathrm{i}\mathbf{k}\cdot\mathbf{R}_i}\, \mathbf{S}_\mu(\mathbf{k})\ . \quad \text{(S5)}$$

The contribution to the energy from the magnetic exchange interactions is

$$\mathcal{H}_\mathrm{J} = -\frac{N}{2}\sum_{\mathbf{k}}\sum_{\mu,\nu}\sum_{\alpha,\beta} S^\alpha_\mu(-\mathbf{k})\, J^{\alpha\beta}_{\mu\nu}(\mathbf{k})\, S^\beta_\nu(\mathbf{k})\ . \quad \text{(S6)}$$

Here the main new quantity is the lattice Fourier transform of the exchange interactions:

$$J^{\alpha\beta}_{\mu\nu}(\mathbf{k}) = \sum_j J^{\alpha\beta}_{i\mu,j\nu}\, e^{\mathrm{i}\mathbf{k}\cdot(\mathbf{R}_j-\mathbf{R}_i)} \implies J^{\alpha\beta}_{\mu\nu}(-\mathbf{k}) = J^{\beta\alpha}_{\nu\mu}(\mathbf{k}) = J^{\alpha\beta}_{\mu\nu}(\mathbf{k})^*\ . \quad \text{(S7)}$$

1. Semiclassical spin dynamics

The semiclassical equation of motion leading to the spin-wave spectrum is given by the Landau-Lifshitz equation (the magnitude of the magnetic moment M_μ has to be included explicitly here):

$$\frac{M_\mu}{\gamma}\frac{\mathrm{d}\mathbf{S}_{i\mu}}{\mathrm{d}t} = \mathbf{B}^{\mathrm{eff}}_{i\mu}(t) \times \mathbf{S}_{i\mu}(t) \ , \tag{S8}$$

$$\mathbf{B}^{\mathrm{eff}}_{i\mu}(t) = -\frac{\partial\mathcal{H}}{\partial\mathbf{S}_{i\mu}(t)} = M_\mu\,\mathbf{B}^{\mathrm{ext}}_{i\mu}(t) + 2K_\mu\cdot\mathbf{S}_{i\mu}(t) + \sum_j\sum_\nu J_{i\mu,j\nu}\cdot\mathbf{S}_{j\nu}(t) \ , \tag{S9}$$

with the gyromagnetic ratio $\gamma = 2$ for spin-only magnetic moments and an additional onsite anisotropy matrix. The idea is to solve its linearized form for a ferromagnetic state, by setting

$$\mathbf{S}_{i\mu}(t) = \hat{\mathbf{z}} + \delta\mathbf{S}_{i\mu}(t) = \hat{\mathbf{z}} + \delta S^x_{i\mu}(t)\,\hat{\mathbf{x}} + \delta S^y_{i\mu}(t)\,\hat{\mathbf{y}} \ . \tag{S10}$$

This gives (to first order in the small time-dependent quantities)

$$\frac{M_\mu}{\gamma}\frac{\mathrm{d}\mathbf{S}_{i\mu}}{\mathrm{d}t} \approx \mathbf{B}^{0,\mathrm{eff}}_{\mu} \times \hat{\mathbf{z}} + \mathbf{B}^{0,\mathrm{eff}}_{i\mu} \times \delta\mathbf{S}_{i\mu}(t) + \delta\mathbf{B}^{\mathrm{eff}}_{i\mu}(t) \times \hat{\mathbf{z}} \ . \tag{S11}$$

The static part has to satisfy the zero torque condition, so:

$$\mathbf{B}^{0,\mathrm{eff}}_{\mu} = B^{0,\mathrm{eff}}_{\mu}\,\hat{\mathbf{z}} \ , \quad B^{0,\mathrm{eff}}_{\mu} = M_\mu B^{0,\mathrm{ext}}_{\mu} + 2K^{zz}_\mu + \sum_\nu J^{zz}_{\mu\nu}(\mathbf{0}) \ . \tag{S12}$$

The time-dependent part is written as a wave with general polarization:

$$\delta\mathbf{S}_{i\mu}(t) = \frac{1}{2}\,\mathrm{Re}\Big[e^{\mathrm{i}(\mathbf{k}\cdot\mathbf{R}_i-\omega t)}\left(\delta S_\mu\cos\alpha_\mu\,e^{\mathrm{i}\phi^+_\mu}\,(\hat{\mathbf{x}}+\mathrm{i}\,\hat{\mathbf{y}}) + \delta S_\mu\sin\alpha_\mu\,e^{\mathrm{i}\phi^-_\mu}\,(\hat{\mathbf{x}}-\mathrm{i}\,\hat{\mathbf{y}})\right)\Big] \tag{S13}$$

$$= \frac{1}{2}\,\mathrm{Re}\big[e^{\mathrm{i}(\mathbf{k}\cdot\mathbf{R}_i-\omega t)}\left(\delta A^+_\mu\mathbf{e}^+ + \delta A^-_\mu\mathbf{e}^-\right)\big] \ , \tag{S14}$$

so the time derivative is

$$\frac{\mathrm{d}\mathbf{S}_{i\mu}}{\mathrm{d}t} = \frac{1}{2}\,\mathrm{Re}\big[-\mathrm{i}\omega\,e^{\mathrm{i}(\mathbf{k}\cdot\mathbf{R}_i-\omega t)}\left(\delta A^+_\mu\mathbf{e}^+ + \delta A^-_\mu\mathbf{e}^-\right)\big] \ . \tag{S15}$$

The first torque term gives

$$\mathbf{B}^{0,\mathrm{eff}}_{\mu} \times \delta\mathbf{S}_{i\mu}(t) = B^{0,\mathrm{eff}}_{\mu}\,\hat{\mathbf{z}} \times \delta\mathbf{S}_{i\mu}(t) = \frac{1}{2}\,\mathrm{Re}\big[-\mathrm{i}B^{0,\mathrm{eff}}_{\mu}\,e^{\mathrm{i}(\mathbf{k}\cdot\mathbf{R}_i-\omega t)}\left(\delta A^+_\mu\mathbf{e}^+ - \delta A^-_\mu\mathbf{e}^-\right)\big] \ . \tag{S16}$$

The second torque term is

$$\delta\mathbf{B}^{\mathrm{eff}}_{i\mu}(t) \times \hat{\mathbf{z}} = \left(M_\mu\delta\mathbf{B}^{\mathrm{ext}}_{i\mu}(t) + 2K_\mu\cdot\delta\mathbf{S}_{i\mu}(t) + \sum_j\sum_\nu J_{i\mu,j\nu}\cdot\delta\mathbf{S}_{j\nu}(t)\right) \times \hat{\mathbf{z}} \ . \tag{S17}$$

The generalized time-dependent magnetic field is written as

$$\delta\mathbf{B}_{i\mu}^{\mathrm{ext}}(t) = \frac{1}{2}\,\mathrm{Re}\big[e^{\mathrm{i}(\mathbf{k}\cdot\mathbf{R}_i-\omega t)}\left(\delta B_\mu^+\mathbf{e}^+ + \delta B_\mu^-\mathbf{e}^-\right)\big] \ , \tag{S18}$$

so

$$M_\mu\delta\mathbf{B}_{i\mu}^{\mathrm{ext}}(t)\times\hat{\mathbf{z}}_\mu = \frac{1}{2}\,\mathrm{Re}\big[+\mathrm{i}M_\mu\, e^{\mathrm{i}(\mathbf{k}\cdot\mathbf{R}_i-\omega t)}\left(\delta B_\mu^+\mathbf{e}^+ - \delta B_\mu^-\mathbf{e}^-\right)\big] \ . \tag{S19}$$

The exchange interactions give

$$\sum_j\sum_\nu J_{i\mu,j\nu}\cdot\delta\mathbf{S}_{j\nu}(t) = \frac{1}{2}\,\mathrm{Re}\left[\sum_j\sum_\nu J_{i\mu,j\nu}\cdot e^{\mathrm{i}(\mathbf{k}\cdot\mathbf{R}_j-\omega t)}\left(\delta A_\nu^+\mathbf{e}^+ + \delta A_\nu^-\mathbf{e}^-\right)\right] \tag{S20}$$

$$\begin{aligned} &= \frac{1}{2}\,\mathrm{Re}\left[e^{\mathrm{i}(\mathbf{k}\cdot\mathbf{R}_i-\omega t)}\sum_\nu\left(J_{\mu\nu}^{++}(\mathbf{k})\,\delta A_\nu^+ + J_{\mu\nu}^{+-}(\mathbf{k})\,\delta A_\nu^-\right)\mathbf{e}^+\right] \\ &+ \frac{1}{2}\,\mathrm{Re}\left[e^{\mathrm{i}(\mathbf{k}\cdot\mathbf{R}_i-\omega t)}\sum_\nu\left(J_{\mu\nu}^{-+}(\mathbf{k})\,\delta A_\nu^+ + J_{\mu\nu}^{--}(\mathbf{k})\,\delta A_\nu^-\right)\mathbf{e}^-\right] \ . \end{aligned} \tag{S21}$$

using

$$\hat{\mathbf{x}} = \frac{1}{2}\left(\mathbf{e}^+ + \mathbf{e}^-\right) \ , \quad \hat{\mathbf{y}} = \frac{\mathrm{i}}{2}\left(\mathbf{e}^- - \mathbf{e}^+\right) \ , \tag{S22}$$

and defining

$$J_{\mu\nu}^{++}(\mathbf{k}) = \frac{1}{2}\left(J_{\mu\nu}^{xx}(\mathbf{k}) + J_{\mu\nu}^{yy}(\mathbf{k}) + \mathrm{i}\,J_{\mu\nu}^{xy}(\mathbf{k}) - \mathrm{i}\,J_{\mu\nu}^{yx}(\mathbf{k})\right) \ , \tag{S23}$$

$$J_{\mu\nu}^{--}(\mathbf{k}) = \frac{1}{2}\left(J_{\mu\nu}^{xx}(\mathbf{k}) + J_{\mu\nu}^{yy}(\mathbf{k}) - \mathrm{i}\,J_{\mu\nu}^{xy}(\mathbf{k}) + \mathrm{i}\,J_{\mu\nu}^{yx}(\mathbf{k})\right) \ , \tag{S24}$$

$$J_{\mu\nu}^{-+}(\mathbf{k}) = \frac{1}{2}\left(J_{\mu\nu}^{xx}(\mathbf{k}) - J_{\mu\nu}^{yy}(\mathbf{k}) + \mathrm{i}\,J_{\mu\nu}^{xy}(\mathbf{k}) + \mathrm{i}\,J_{\mu\nu}^{yx}(\mathbf{k})\right) \ , \tag{S25}$$

$$J_{\mu\nu}^{+-}(\mathbf{k}) = \frac{1}{2}\left(J_{\mu\nu}^{xx}(\mathbf{k}) - J_{\mu\nu}^{yy}(\mathbf{k}) - \mathrm{i}\,J_{\mu\nu}^{xy}(\mathbf{k}) - \mathrm{i}\,J_{\mu\nu}^{yx}(\mathbf{k})\right) \ . \tag{S26}$$

The same structure applies to the onsite magnetic anisotropy. The actual torque follows along the lines of the magnetic field part.

Comparing terms we get the linearized equation for the spin dynamics:

$$\frac{\omega M_\mu}{\gamma}\begin{pmatrix}\delta A_\mu^+\\ \delta A_\mu^-\end{pmatrix} = \sum_\nu\begin{pmatrix}B_\mu^{0,\mathrm{eff}}\delta_{\mu\nu} - J_{\mu\nu}^{++}(\mathbf{k}) & -J_{\mu\nu}^{+-}(\mathbf{k})\\ J_{\mu\nu}^{-+}(\mathbf{k}) & -B_\mu^{0,\mathrm{eff}}\delta_{\mu\nu} + J_{\mu\nu}^{--}(\mathbf{k})\end{pmatrix}\begin{pmatrix}\delta A_\nu^+\\ \delta A_\nu^-\end{pmatrix} - M_\mu\begin{pmatrix}\delta B_\mu^+\\ -\delta B_\mu^-\end{pmatrix} \ . \tag{S27}$$

2. Eigenvalues and eigenvectors of the dynamical matrix

Forgetting about the driving magnetic field for the moment and introducing a symmetrizing transformation,

$$\begin{pmatrix} \delta A^+_\mu \\ \delta A^-_\mu \end{pmatrix} = \sqrt{\frac{\gamma}{M_\mu}} \begin{pmatrix} \delta \widetilde{A}^+_\mu \\ \delta \widetilde{A}^-_\mu \end{pmatrix} , \tag{S28}$$

we arrive at

$$\omega \begin{pmatrix} \delta \widetilde{A}^+_\mu \\ \delta \widetilde{A}^-_\mu \end{pmatrix} = \sum_\nu \begin{pmatrix} \frac{\gamma B^{0,\mathrm{eff}}_\mu}{M_\mu} \delta_{\mu\nu} - \frac{\gamma J^{++}_{\mu\nu}(\mathbf{k})}{\sqrt{M_\mu M_\nu}} & -\frac{\gamma J^{+-}_{\mu\nu}(\mathbf{k})}{\sqrt{M_\mu M_\nu}} \\ \frac{\gamma J^{-+}_{\mu\nu}(\mathbf{k})}{\sqrt{M_\mu M_\nu}} & -\frac{\gamma B^{0,\mathrm{eff}}_\mu}{M_\mu} \delta_{\mu\nu} + \frac{\gamma J^{--}_{\mu\nu}(\mathbf{k})}{\sqrt{M_\mu M_\nu}} \end{pmatrix} \begin{pmatrix} \delta \widetilde{A}^+_\nu \\ \delta \widetilde{A}^-_\nu \end{pmatrix} , \tag{S29}$$

or

$$\omega \begin{pmatrix} \delta \widetilde{A}^+_\mu \\ \delta \widetilde{A}^-_\mu \end{pmatrix} = \sum_\nu \begin{pmatrix} D^{++}_{\mu\nu}(\mathbf{k}) & D^{+-}_{\mu\nu}(\mathbf{k}) \\ D^{-+}_{\mu\nu}(\mathbf{k}) & D^{--}_{\mu\nu}(\mathbf{k}) \end{pmatrix} \begin{pmatrix} \delta \widetilde{A}^+_\nu \\ \delta \widetilde{A}^-_\nu \end{pmatrix} , \tag{S30}$$

which defines a non-hermitian eigenvalue problem. We can define right eigenvectors

$$\sum_\nu \begin{pmatrix} D^{++}_{\mu\nu}(\mathbf{k}) & D^{+-}_{\mu\nu}(\mathbf{k}) \\ D^{-+}_{\mu\nu}(\mathbf{k}) & D^{--}_{\mu\nu}(\mathbf{k}) \end{pmatrix} \begin{pmatrix} R^+_{n,\nu}(\mathbf{k}) \\ R^-_{n,\nu}(\mathbf{k}) \end{pmatrix} = \omega_n(\mathbf{k}) \begin{pmatrix} R^+_{n,\mu}(\mathbf{k}) \\ R^-_{n,\mu}(\mathbf{k}) \end{pmatrix} , \tag{S31}$$

and left eigenvectors

$$\sum_\mu \begin{pmatrix} L^+_{n,\mu}(\mathbf{k}) & L^-_{n,\mu}(\mathbf{k}) \end{pmatrix} \begin{pmatrix} D^{++}_{\mu\nu}(\mathbf{k}) & D^{+-}_{\mu\nu}(\mathbf{k}) \\ D^{-+}_{\mu\nu}(\mathbf{k}) & D^{--}_{\mu\nu}(\mathbf{k}) \end{pmatrix} = \begin{pmatrix} L^+_{n,\nu}(\mathbf{k}) & L^-_{n,\nu}(\mathbf{k}) \end{pmatrix} \omega_n(\mathbf{k}) , \tag{S32}$$

such that

$$\sum_{\mu,\nu} \begin{pmatrix} L^+_{n,\mu}(\mathbf{k}) & L^-_{n,\mu}(\mathbf{k}) \end{pmatrix} \begin{pmatrix} D^{++}_{\mu\nu}(\mathbf{k}) & D^{+-}_{\mu\nu}(\mathbf{k}) \\ D^{-+}_{\mu\nu}(\mathbf{k}) & D^{--}_{\mu\nu}(\mathbf{k}) \end{pmatrix} \begin{pmatrix} R^+_{n,\nu}(\mathbf{k}) \\ R^-_{n,\nu}(\mathbf{k}) \end{pmatrix} = \omega_n(\mathbf{k})\, C_n(\mathbf{k}) . \tag{S33}$$

We can impose the normalization

$$\sum_\mu \left(L^+_{n,\mu}(\mathbf{k}) R^+_{m,\mu}(\mathbf{k}) + L^-_{n,\mu}(\mathbf{k}) R^-_{m,\mu}(\mathbf{k}) \right) = C_n(\mathbf{k})\, \delta_{nm} = \delta_{nm} . \tag{S34}$$

Conversely (the sum is over all positive and negative frequencies),

$$\sum_n \begin{pmatrix} R^+_{n,\mu}(\mathbf{k}) L^+_{n,\nu}(\mathbf{k}) & R^+_{n,\mu}(\mathbf{k}) L^-_{n,\nu}(\mathbf{k}) \\ R^-_{n,\mu}(\mathbf{k}) L^+_{n,\nu}(\mathbf{k}) & R^-_{n,\mu}(\mathbf{k}) L^-_{n,\nu}(\mathbf{k}) \end{pmatrix} = \delta_{\mu\nu} \begin{pmatrix} 1 & 0 \\ 0 & 1 \end{pmatrix} . \tag{S35}$$

Lastly, the generalized eigendecomposition is

$$\begin{pmatrix} D^{++}_{\mu\nu}(\mathbf{k}) & D^{+-}_{\mu\nu}(\mathbf{k}) \\ D^{-+}_{\mu\nu}(\mathbf{k}) & D^{--}_{\mu\nu}(\mathbf{k}) \end{pmatrix} = \sum_n \omega_n(\mathbf{k}) \begin{pmatrix} R^+_{n,\mu}(\mathbf{k}) L^+_{n,\nu}(\mathbf{k}) & R^+_{n,\mu}(\mathbf{k}) L^-_{n,\nu}(\mathbf{k}) \\ R^-_{n,\mu}(\mathbf{k}) L^+_{n,\nu}(\mathbf{k}) & R^-_{n,\mu}(\mathbf{k}) L^-_{n,\nu}(\mathbf{k}) \end{pmatrix} . \tag{S36}$$

3. Dynamical magnetic susceptibility

The generalized dynamical magnetic susceptibility is defined as

$$\delta \mathbf{S}_{i\mu}(t) = \frac{1}{2}\,\mathrm{Re}\big[e^{\mathrm{i}(\mathbf{k}\cdot\mathbf{R}_i-\omega t)}\left(\delta A^+_\mu \mathbf{e}^+ + \delta A^-_\mu \mathbf{e}^-\right)\big] \tag{S37}$$

$$= \sum_j \sum_\nu \int_{-\infty}^{t} \mathrm{d}t'\; \chi_{i\mu,j\nu}(t-t') \cdot \delta \mathbf{B}^{\mathrm{ext}}_{j\nu}(t') \tag{S38}$$

$$= \frac{1}{2}\,\mathrm{Re}\left[e^{\mathrm{i}(\mathbf{k}\cdot\mathbf{R}_i-\omega t)} \sum_\nu \chi_{\mu\nu}(\mathbf{k},\omega+\mathrm{i}0)\cdot\left(\delta B^+_\nu(\mathbf{k},\omega)\,\mathbf{e}^+ + \delta B^-_\nu(\mathbf{k},\omega)\,\mathbf{e}^-\right)\right] , \tag{S39}$$

so that it gives the connection

$$\begin{pmatrix} \delta A^+_\mu(\mathbf{k},\omega) \\ \delta A^-_\mu(\mathbf{k},\omega) \end{pmatrix} = \sum_\nu \begin{pmatrix} \chi^{++}_{\mu\nu}(\mathbf{k},\omega+\mathrm{i}0) & \chi^{+-}_{\mu\nu}(\mathbf{k},\omega+\mathrm{i}0) \\ \chi^{-+}_{\mu\nu}(\mathbf{k},\omega+\mathrm{i}0) & \chi^{--}_{\mu\nu}(\mathbf{k},\omega+\mathrm{i}0) \end{pmatrix} \begin{pmatrix} \delta B^+_\nu(\mathbf{k},\omega) \\ \delta B^-_\nu(\mathbf{k},\omega) \end{pmatrix} . \tag{S40}$$

Reintroducing the driving field,

$$\sum_\nu \begin{pmatrix} D^{++}_{\mu\nu}(\mathbf{k}) - \omega\,\delta_{\mu\nu} & D^{+-}_{\mu\nu}(\mathbf{k}) \\ D^{-+}_{\mu\nu}(\mathbf{k}) & D^{--}_{\mu\nu}(\mathbf{k}) - \omega\,\delta_{\mu\nu} \end{pmatrix} \begin{pmatrix} \delta \widetilde{A}^+_\nu \\ \delta \widetilde{A}^-_\nu \end{pmatrix} = \sqrt{\gamma M_\mu} \begin{pmatrix} 1 & 0 \\ 0 & -1 \end{pmatrix} \begin{pmatrix} \delta B^+_\mu \\ \delta B^-_\mu \end{pmatrix} , \tag{S41}$$

and going back to the unscaled amplitudes

$$\begin{pmatrix} \delta A^+_\mu \\ \delta A^-_\mu \end{pmatrix} = \sum_\nu \sum_n \frac{\sqrt{M_\mu M_\nu}}{\omega_n(\mathbf{k}) - \omega - \mathrm{i}0} \begin{pmatrix} R^+_{n,\mu}(\mathbf{k})L^+_{n,\nu}(\mathbf{k}) & -R^+_{n,\mu}(\mathbf{k})L^-_{n,\nu}(\mathbf{k}) \\ R^-_{n,\mu}(\mathbf{k})L^+_{n,\nu}(\mathbf{k}) & -R^-_{n,\mu}(\mathbf{k})L^-_{n,\nu}(\mathbf{k}) \end{pmatrix} \begin{pmatrix} \delta B^+_\nu \\ \delta B^-_\nu \end{pmatrix} . \tag{S42}$$

The dynamical magnetic susceptibility is thus given by (zero temperature)

$$\chi_{\mu\nu}(\mathbf{k},\omega+\mathrm{i}0) = \sum_n \frac{\sqrt{M_\mu M_\nu}}{\omega_n(\mathbf{k}) - \omega - \mathrm{i}0} \begin{pmatrix} R^+_{n,\mu}(\mathbf{k})L^+_{n,\nu}(\mathbf{k}) & -R^+_{n,\mu}(\mathbf{k})L^-_{n,\nu}(\mathbf{k}) \\ R^-_{n,\mu}(\mathbf{k})L^+_{n,\nu}(\mathbf{k}) & -R^-_{n,\mu}(\mathbf{k})L^-_{n,\nu}(\mathbf{k}) \end{pmatrix} . \tag{S43}$$

4. Dynamical structure factor

The well-known van Hove formula gives the dynamical structure factor $\mathcal{S}(\mathbf{k},\omega)$, where $\mathbf{k}$ is the change in wavevector between the incoming and the scattered neutrons and ω is the corresponding energy loss. $\mathcal{S}(\mathbf{k},\omega)$ is proportional to the imaginary part of the dynamical susceptibility, so in the current notation this reads:

$$\mathcal{S}(\mathbf{k},\omega) \propto \sum_{\mu,\nu} e^{\mathrm{i}\mathbf{k}\cdot(\mathbf{R}_\mu-\mathbf{R}_\nu)}\,\mathrm{Tr}\mathcal{M}(\mathbf{k})\,\rho_{\mu\nu}(\mathbf{k},\omega) . \tag{S44}$$

The first quantity that appears in this formula is a phase factor arising from the distance between magnetic atoms inside a given unit cell, $\mathbf{R}_\mu - \mathbf{R}_\nu$. The second quantity $\mathcal{M}^{\alpha\beta}(\mathbf{k})$ takes into account that the neutron spin interacts with the magnetic field created by the magnons in a purely transverse way. For unpolarized neutron scattering ($\alpha, \beta = x, y$ for ferromagnet along z)

$$\mathcal{M}^{\alpha\beta}(\mathbf{k}) = \delta^{\alpha\beta} - \frac{k^\alpha k^\beta}{k^2} \ . \tag{S45}$$

The third quantity in Eq. (S44) is $\rho_{\mu\nu}(\mathbf{k}, \omega)$, the magnon spectral density which according to the dynamical susceptibility is

$$\rho_{\mu\nu}(\mathbf{k}, \omega) = \sum_n f_n(\mathbf{k}, \omega) \sqrt{M_\mu M_\nu}\, U \begin{pmatrix} R^+_{n,\mu}(\mathbf{k}) L^+_{n,\nu}(\mathbf{k}) & -R^+_{n,\mu}(\mathbf{k}) L^-_{n,\nu}(\mathbf{k}) \\ R^-_{n,\mu}(\mathbf{k}) L^+_{n,\nu}(\mathbf{k}) & -R^-_{n,\mu}(\mathbf{k}) L^-_{n,\nu}(\mathbf{k}) \end{pmatrix} U^\dagger \ . \tag{S46}$$

This quantity is projected on the different pairs of magnetic atoms in the unit cell, μ and ν. We can include broadening for each magnon mode, $f_n(\mathbf{k}, \omega)$, for example given by the damped harmonic oscillator form using the magnon energies $\omega_n(\mathbf{k})$ and damping rate η

$$f_n(\mathbf{k}, \omega) = \frac{\eta\omega}{(\omega - \omega_n(\mathbf{k}))^2 + \eta^2} \ \underset{\eta \to 0}{\longrightarrow} \ \delta(\omega - \omega_n(\mathbf{k})) \ . \tag{S47}$$

The factor $\sqrt{M_\mu M_\nu}$ takes into account that different atoms can have different magnetic moments and so scatter neutrons with different efficiencies; a more rigorous expression would use instead the atomic magnetic form factors here. The transformation matrix from $(+, -)$ to (x, y) components is given by

$$U = \begin{pmatrix} \hat{\mathbf{x}} \cdot \mathbf{e}^+ & \hat{\mathbf{x}} \cdot \mathbf{e}^- \\ \hat{\mathbf{y}} \cdot \mathbf{e}^+ & \hat{\mathbf{y}} \cdot \mathbf{e}^- \end{pmatrix} = \begin{pmatrix} 1 & 1 \\ \mathrm{i} & -\mathrm{i} \end{pmatrix} \ , \tag{S48}$$

The transformation matrices act as

$$\begin{pmatrix} A^{xx} & A^{xy} \\ A^{yx} & A^{yy} \end{pmatrix} = \begin{pmatrix} 1 & 1 \\ \mathrm{i} & -\mathrm{i} \end{pmatrix} \begin{pmatrix} A^{++} & -A^{+-} \\ A^{-+} & -A^{--} \end{pmatrix} \begin{pmatrix} 1 & -\mathrm{i} \\ 1 & \mathrm{i} \end{pmatrix} \tag{S49}$$

$$= \begin{pmatrix} A^{++} - A^{--} - A^{+-} + A^{-+} & -\mathrm{i}\,(A^{++} + A^{--} + A^{+-} + A^{-+}) \\ \mathrm{i}\,(A^{++} + A^{--} - A^{+-} - A^{-+}) & A^{++} - A^{--} + A^{+-} - A^{-+} \end{pmatrix} \ . \tag{S50}$$

The middle matrix in Eq. (S44) contains the left and right eigenvectors obtained in the process of solving Eq. (S29) and describing the actual precession motion of the different magnetic moments.

D. Theoretical dependence of the magnon bands on the structural parameters

Here we show the results of a systematic theoretical investigation of how the magnon bands depend on small distortions of the experimental crystal. For each chosen pair of values for a and c, the internal atomic positions were optimized with Quantum Espresso, followed by the calculation of the magnetic interactions with juKKR. Lastly, the magnon energies were computed according to Eq. (S29).

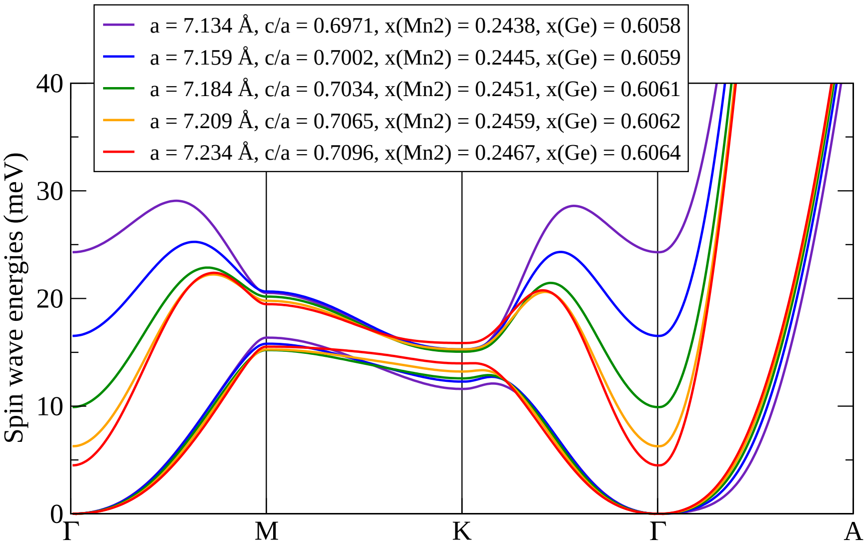

Fig. S12. **Theoretical evolution of the spin wave energies of Mn_5Ge_3 under hydrostatic pressure.**

The results for a uniform volume expansion/contraction ("hydrostatic pressure") are shown in Fig. S12 and for a uniaxial deformation with constant volume ("uniaxial pressure") are shown in Fig. S13. We see that the lowest-energy magnon branch is rather insensitive to the various structural deformations, except for the $\mathrm{M} - \mathrm{K}$ path under uniaxial pressure. The higher magnon branch is rather insensitive to hydrostatic pressure for the $\mathrm{M} - \mathrm{K}$ path, but the way it disperses away from Γ is heavily modified by structural deformations.

Two important remarks can be made using these results. Firstly, the gap between the two magnon branches at the K-point never closes, as the Dzyaloshinskii-Moriya interaction never vanishes, and so is a qualitatively robust feature. Secondly, the good agreement between theory and experiment concerning the Curie temperature reported in Ref. [25], the good agreement between the computed magnetic interactions in Ref. [25] and in our work, and the quantitative disagreement between the theoretically and experimentally determined

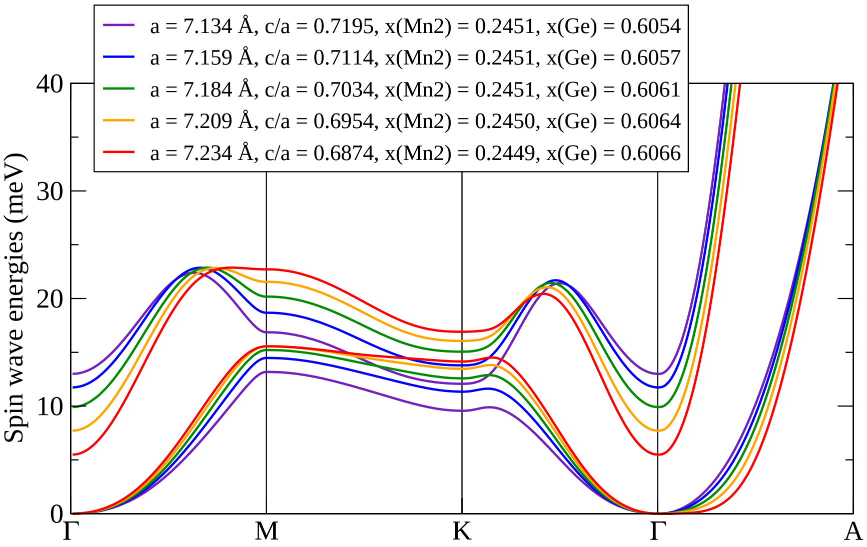

Fig. S13. **Theoretical evolution of the spin wave energies of Mn_5Ge_3 under uniaxial pressure.**

magnon energies can be reconciled. We propose that the magnitude of the Curie temperature is set by the spin stiffness of the lowest-energy magnon branch, which is quite robust against structural deformations and for which the measured spin wave energies in the $\Gamma - \mathrm{A}$ direction are in good agreement with our calculations.

E. Simplified effective spin model

The spin model parameterized using the magnetic interactions obtained from the DFT calculations was found to be a poor quantitative description of the experimentally measured magnon bands in the $\Gamma - \mathrm{M} - \mathrm{K} - \Gamma$ path, so we decided to construct a simplified model that could be used to gain understanding about the underlying physics. The structure of the magnetic sublattices of Mn_5Ge_3 is shown again in Fig. S14(a). Within the unit cell, the Mn1 atoms form two different chains along the c-axis; in the ab-plane these chains are arranged with respect to each other in a honeycomb lattice. The Mn2 atoms form a column of face-sharing octahedra. Both theory and experiment (from the measured dispersion along $\Gamma - \mathrm{A}$) agree that the strongest magnetic interactions are along the c-axis and should have a predominantly ferromagnetic nature. Another observation which is relevant for constructing the simplified model is that only three magnon modes are observed in the $\Gamma - \mathrm{M} - \mathrm{K} - \Gamma$

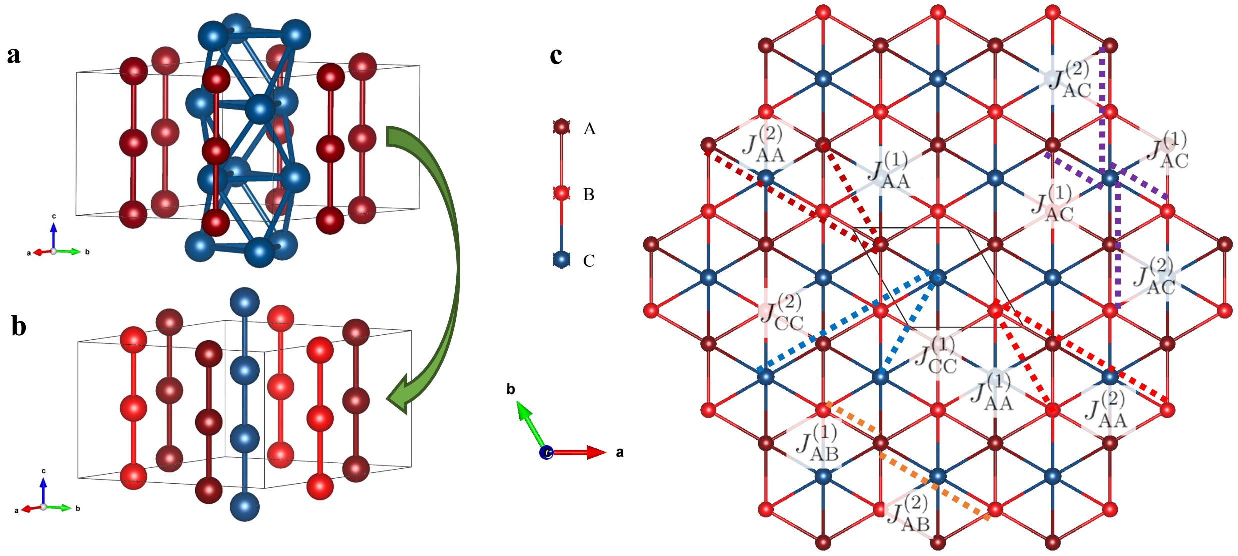

Fig. S14. **Construction of the simplified effective spin model.** **a** The original magnetic sublattices. **b** The simplified magnetic sublattices after Mn2 trimers are replaced with an effective spin. **c** Definition of the magnetic interactions for the effective model (top view). The Mn1 sites form the A and B sublattices, and the effective Mn2 spin forms the C sublattice. The unit cell is indicated by the thin black lines.

path, in the accessible energy range. This suggests to assign one degree of freedom to each of the two Mn1 chains, and the third to the Mn2 octahedra, leading to the simplified effective magnetic sublattices shown in Fig. S14(b); the length of the unit cell along the c-axis is then half of the original value. This is further justified by comparing our results with the magnon dispersion of the isostructural $MnFe_4Si_3$ where magnetic moments occur only in the Mn2 sites, resulting in one in-plane dispersion in the investigated energy range [27].

The effective spin model is thus specified by the three magnetic sublattices

$$\mathbf{R}_{\mathrm{A}} = \frac{1}{3}\mathbf{a}_1 + \frac{2}{3}\mathbf{a}_2 \ , \quad \mathbf{R}_{\mathrm{B}} = \frac{2}{3}\mathbf{a}_1 + \frac{1}{3}\mathbf{a}_2 \ , \quad \mathbf{R}_{\mathrm{C}} = \frac{1}{2}\mathbf{a}_3 \ , \tag{S51}$$

in terms of the hexagonal unit cell vectors of Mn_5Ge_3; $|\mathbf{a}_3| = c/2$ as explained in the previous paragraph. We first consider the set of Heisenberg exchange interactions shown in Fig. S14(c). The interaction strength for each shell is denoted $J^{(n)}_{\mu\nu}$. By symmetry we have $J^{(n)}_{\mathrm{AA}} = J^{(n)}_{\mathrm{BB}}$ and $J^{(n)}_{\mathrm{AC}} = J^{(n)}_{\mathrm{CA}} = J^{(n)}_{\mathrm{BC}} = J^{(n)}_{\mathrm{CB}}$. The list of parameters by increasing bond distance is then

$$J^{(1)}_{\mathrm{AB}} \ , \quad J^{(1)}_{\mathrm{AC}} \ , \quad J^{(1)}_{\mathrm{AA}} \ , \quad J^{(1)}_{\mathrm{CC}} \ , \quad J^{(2)}_{\mathrm{AB}} \ , \quad J^{(2)}_{\mathrm{AC}} \ , \quad J^{(2)}_{\mathrm{AA}} \ , \quad J^{(2)}_{\mathrm{CC}} \ . \tag{S52}$$

Expressing the wave vector in terms of the reciprocal lattice basis vectors,

$$\mathbf{q} = q_1\mathbf{b}_1 + q_2\mathbf{b}_2 + q_3\mathbf{b}_3 \ , \quad \mathbf{a}_i \cdot \mathbf{b}_j = \delta_{ij} \ , \tag{S53}$$

we need to compute the lattice Fourier transform of the magnetic interactions to obtain

$$J(\mathbf{q}) = \begin{pmatrix} J_{\mathrm{AA}}(\mathbf{q}) & J_{\mathrm{AB}}(\mathbf{q}) & J_{\mathrm{AC}}(\mathbf{q}) \\ J_{\mathrm{AB}}(\mathbf{q})^* & J_{\mathrm{AA}}(\mathbf{q}) & J_{\mathrm{BC}}(\mathbf{q}) \\ J_{\mathrm{AC}}(\mathbf{q})^* & J_{\mathrm{BC}}(\mathbf{q})^* & J_{\mathrm{CC}}(\mathbf{q}) \end{pmatrix} \ . \tag{S54}$$

The required expressions are (setting $q_3 = 0$):

$$\begin{aligned} J_{\mu\mu}(\mathbf{q}) &= 2J^{(1)}_{\mu\mu}\left(\cos 2\pi q_1 + \cos 2\pi q_2 + \cos 2\pi(q_1+q_2)\right) \\ &\quad + 2J^{(2)}_{\mu\mu}\left(\cos 2\pi(q_1-q_2) + \cos 2\pi(2q_1+q_2) + \cos 2\pi(q_1+2q_2)\right) \ , && \text{(S55)} \\ J_{\mathrm{AB}}(\mathbf{q}) &= J^{(1)}_{\mathrm{AB}}\left(1 + \cos 2\pi q_1 + \cos 2\pi q_2\right) - \mathrm{i}\, J^{(1)}_{\mathrm{AB}}\left(\sin 2\pi q_1 - \sin 2\pi q_2\right) \\ &\quad + J^{(2)}_{\mathrm{AB}}\left(2\cos 2\pi(q_1+q_2) + \cos 2\pi(q_1-q_2)\right) - \mathrm{i}\, J^{(2)}_{\mathrm{AB}} \sin 2\pi(q_1-q_2) \ , && \text{(S56)} \\ J_{\mathrm{AC}}(\mathbf{q}) &= J^{(1)}_{\mathrm{AC}}\left(1 + \cos 2\pi q_2 + \cos 2\pi(q_1+q_2)\right) + \mathrm{i}\, J^{(1)}_{\mathrm{AC}}\left(\sin 2\pi q_2 + \sin 2\pi(q_1+q_2)\right) \\ &\quad + J^{(2)}_{\mathrm{AC}}\left(2\cos 2\pi q_1 + \cos 2\pi(q_1+2q_2)\right) + \mathrm{i}\, J^{(1)}_{\mathrm{AC}} \sin 2\pi(q_1+2q_2) && \text{(S57)} \\ J_{\mathrm{BC}}(\mathbf{q}) &= J^{(1)}_{\mathrm{AC}}\left(1 + \cos 2\pi q_1 + \cos 2\pi(q_1+q_2)\right) + \mathrm{i}\, J^{(1)}_{\mathrm{AC}}\left(\sin 2\pi q_1 + \sin 2\pi(q_1+q_2)\right) \\ &\quad + J^{(2)}_{\mathrm{AC}}\left(2\cos 2\pi q_2 + \cos 2\pi(q_1+2q_2)\right) + \mathrm{i}\, J^{(1)}_{\mathrm{AC}} \sin 2\pi(2q_1+q_2) \ . && \text{(S58)} \end{aligned}$$

At Γ this gives

$$J(\mathbf{0}) = \begin{pmatrix} 6\left(J^{(1)}_{\mathrm{AA}} + J^{(2)}_{\mathrm{AA}}\right) & 3\left(J^{(1)}_{\mathrm{AB}} + J^{(2)}_{\mathrm{AB}}\right) & 3\left(J^{(1)}_{\mathrm{AC}} + J^{(2)}_{\mathrm{AC}}\right) \\ 3\left(J^{(1)}_{\mathrm{AB}} + J^{(2)}_{\mathrm{AB}}\right) & 6\left(J^{(1)}_{\mathrm{AA}} + J^{(2)}_{\mathrm{AA}}\right) & 3\left(J^{(1)}_{\mathrm{AC}} + J^{(2)}_{\mathrm{AC}}\right) \\ 3\left(J^{(1)}_{\mathrm{AC}} + J^{(2)}_{\mathrm{AC}}\right) & 3\left(J^{(1)}_{\mathrm{AC}} + J^{(2)}_{\mathrm{AC}}\right) & 6\left(J^{(1)}_{\mathrm{CC}} + J^{(2)}_{\mathrm{CC}}\right) \end{pmatrix} \ . \tag{S59}$$

The spin wave energies are obtained from the dynamical matrix (c.f. Eq. (S29); the additional prefactor of 2 is to compare with the convention used for the values in Table I of the main text)

$$D(\mathbf{q}) = 2\gamma \begin{pmatrix} \frac{B_{\mathrm{A}} - J_{\mathrm{AA}}(\mathbf{q})}{M_{\mathrm{A}}} & -\frac{J_{\mathrm{AB}}(\mathbf{q})}{M_{\mathrm{A}}} & -\frac{J_{\mathrm{AC}}(\mathbf{q})}{\sqrt{M_{\mathrm{A}}M_{\mathrm{C}}}} \\ -\frac{J_{\mathrm{AB}}(\mathbf{q})^*}{M_{\mathrm{A}}} & \frac{B_{\mathrm{A}} - J_{\mathrm{AA}}(\mathbf{q})}{M_{\mathrm{A}}} & -\frac{J_{\mathrm{BC}}(\mathbf{q})}{\sqrt{M_{\mathrm{A}}M_{\mathrm{C}}}} \\ -\frac{J_{\mathrm{AC}}(\mathbf{q})^*}{\sqrt{M_{\mathrm{A}}M_{\mathrm{C}}}} & -\frac{J_{\mathrm{BC}}(\mathbf{q})^*}{\sqrt{M_{\mathrm{A}}M_{\mathrm{C}}}} & \frac{B_{\mathrm{C}} - J_{\mathrm{CC}}(\mathbf{q})}{M_{\mathrm{C}}} \end{pmatrix} \ . \tag{S60}$$

with $\gamma = 2$, $M_\mathrm{A} = 2S_\mathrm{A} = 2$, $M_\mathrm{C} = 2S_\mathrm{C} = 9$ (recall that three Mn2 atoms were grouped into one effective spin), and

$$B_\mathrm{A} = J_\mathrm{AA}(\mathbf{0}) + J_\mathrm{AB}(\mathbf{0}) + J_\mathrm{AC}(\mathbf{0}) \ , \quad B_\mathrm{C} = J_\mathrm{CC}(\mathbf{0}) + 2J_\mathrm{AC}(\mathbf{0}) \ . \tag{S61}$$

To extract the required magnetic interaction parameters, we focus on the $\Gamma - \mathrm{M} - \mathrm{K} - \Gamma$ path (hence $q_3 = 0$) and diagonalize the spin wave matrix at the high-symmetry points. The eigenvalues at Γ are

$$\omega_1(\Gamma) = 0 \ , \quad \omega_2(\Gamma) = \frac{26(J^{(1)}_\mathrm{AC} + J^{(2)}_\mathrm{AC})}{3} \ , \tag{S62}$$

$$\omega_3(\Gamma) = 6(2J^{(1)}_\mathrm{AB} + 2J^{(2)}_\mathrm{AB} + J^{(1)}_\mathrm{AC} + J^{(2)}_\mathrm{AC}) \ . \tag{S63}$$

The eigenvalues at M are

$$\omega_1(\mathrm{M}) = 2\left(8\big(J^{(1)}_\mathrm{AA} + J^{(2)}_\mathrm{AA}\big) + 2\big(J^{(1)}_\mathrm{AB} + 3J^{(2)}_\mathrm{AB}\big) + 3\big(J^{(1)}_\mathrm{AC} + J^{(2)}_\mathrm{AC}\big)\right) \ , \tag{S64}$$

$$9\omega_\pm(\mathrm{M}) = 72(J^{(1)}_\mathrm{AA} + J^{(2)}_\mathrm{AA}) + 36J^{(1)}_\mathrm{AB} + 39(J^{(1)}_\mathrm{AC} + J^{(2)}_\mathrm{AC}) + 16(J^{(1)}_\mathrm{CC} + J^{(2)}_\mathrm{CC}) \pm \sqrt{X} \ , \tag{S65}$$

with

$$\begin{aligned} X = &\left(72(J^{(1)}_\mathrm{AA} + J^{(2)}_\mathrm{AA}) + 36J^{(1)}_\mathrm{AB} + 39(J^{(1)}_\mathrm{AC} + J^{(2)}_\mathrm{AC}) + 16(J^{(1)}_\mathrm{CC} + J^{(2)}_\mathrm{CC})\right)^2 \\ &+ 4\left(-432\big((2J^{(1)}_\mathrm{AA} + J^{(2)}_\mathrm{AA}) + J^{(1)}_\mathrm{AB} + J^{(1)}_\mathrm{CC} + J^{(2)}_\mathrm{CC}\big)(J^{(1)}_\mathrm{AC} + J^{(2)}_\mathrm{AC}) - 864J^{(1)}_\mathrm{AC}J^{(2)}_\mathrm{AC} - 288(J^{(1)}_\mathrm{AC})^2 \right. \\ &\left. - 576\big(2(J^{(1)}_\mathrm{AA} + J^{(2)}_\mathrm{AA}) + J^{(1)}_\mathrm{AB}\big)(J^{(1)}_\mathrm{CC} + J^{(2)}_\mathrm{CC})\right) \ . \end{aligned} \tag{S66}$$

The eigenvalues at K are ($\omega_1(\mathrm{K})$ is doubly degenerate)

$$\omega_1(\mathrm{K}) = 6\left(3J^{(1)}_\mathrm{AA} + J^{(1)}_\mathrm{AB} + J^{(2)}_\mathrm{AB} + J^{(1)}_\mathrm{AC} + J^{(2)}_\mathrm{AC}\right) \ , \tag{S67}$$

$$\omega_2(\mathrm{K}) = \frac{4\left(2\big(J^{(1)}_\mathrm{AC} + J^{(2)}_\mathrm{AC}\big) + 3J^{(1)}_\mathrm{CC}\right)}{3} \ . \tag{S68}$$

To have a set of 8 equations for 8 unknowns, we add one of the eigenvalues at K/2 (halfway between K and Γ)

$$\omega_1(\mathrm{K}/2) = 2\left(5\big(J^{(1)}_\mathrm{AA} + J^{(1)}_\mathrm{AB}\big) + 8J^{(2)}_\mathrm{AA} + 3\big(J^{(2)}_\mathrm{AB} + J^{(1)}_\mathrm{AC} + J^{(2)}_\mathrm{AC}\big)\right) \ . \tag{S69}$$

We then solve the following set of equations:

$$\begin{aligned} &\omega_2(\Gamma) = 55\,\mathrm{meV} \ , \quad \omega_3(\Gamma) = 5\,\mathrm{meV} \ , \quad \omega_1(\mathrm{M}) = 8\,\mathrm{meV} \ , \quad \omega_-(\mathrm{M}) = 15\,\mathrm{meV} \ , \\ &\omega_+(\mathrm{M}) = 21\,\mathrm{meV} \ , \quad \omega_1(\mathrm{K}) = 11\,\mathrm{meV} \ , \quad \omega_2(\mathrm{K}) = 19\,\mathrm{meV} \ , \quad \omega_1(\mathrm{K}/2) = 11\,\mathrm{meV} \ . \end{aligned} \tag{S70}$$

Most chosen values are related to the measured magnon energies at the high-symmetry points, but rounded. The chosen value for the lowest energy at K is the midpoint of the experimental magnon gap; the values at Γ were adjusted to make the magnon bands pass as close as possible through the experimental data on the $\Gamma - \mathrm{M}$ path.

This results in the following set of interactions:

$$J^{(1)}_{\mathrm{AB}} = -1.421\,\mathrm{meV}\ , \quad J^{(1)}_{\mathrm{AC}} = 5.318\,\mathrm{meV}\ , \quad J^{(1)}_{\mathrm{AA}} = -0.585\,\mathrm{meV}\ , \quad J^{(1)}_{\mathrm{CC}} = 0.519\,\mathrm{meV}\ ,$$
$$J^{(2)}_{\mathrm{AB}} = -1.335\,\mathrm{meV}\ , \quad J^{(2)}_{\mathrm{AC}} = 1.028\,\mathrm{meV}\ , \quad J^{(2)}_{\mathrm{AA}} = 0.063\,\mathrm{meV}\ , \quad J^{(2)}_{\mathrm{CC}} = -0.313\,\mathrm{meV}\ . \tag{S71}$$

We see that the dominant FM interactions are between Mn1 and Mn2 (AC) — the strong Mn1-Mn1 FM interactions only affect the dispersion on the $\Gamma - \mathrm{A}$ path and so do not affect these results —, while the coupling between the two Mn1 chains (AB) is AFM. This is in qualitative agreement with the theoretical values reported in Table I of the main text; a direct numerical comparison is not possible because the effective spin model is mapping the magnon energies to a set of 8 parameters, while the calculated magnetic interactions from DFT are long-ranged and only the first few values are given in the table.

There are two additional interactions to be added to the model, in order to obtain the magnon gap at K and to have a better description of the dispersion along $\Gamma - \mathrm{A}$, as shown in Fig. 2(b) of the main text. The DMI is needed to obtain the magnon gap at K, and we include the simplest one that is consistent with the symmetry and with the simplified set of magnetic sublattices. We set the DMI vector along the c-axis with magnitude $D^{(1)}_{\mathrm{AA}} = 0.15\,\mathrm{meV}$ and enforce C_{3v} symmetry around each A site; the pattern of DMI vectors around each B site is the opposite of the one for the A sites. For an improved dispersion along $\Gamma - \mathrm{A}$, we add the nearest-neighbor intra-chain interactions along the c-axis, setting $J^{(c)}_{\mathrm{AA}} = 15\,\mathrm{meV}$ and $J^{(c)}_{\mathrm{CC}} = 25\,\mathrm{meV}$.

F. Simulated INS using the effective spin model

Lastly, we consider the impact of the constructive or destructive interference on the magnon bands which are expected to be observable in INS. The results shown in Fig. S15 make use of the effective spin model of the previous section, but the same conclusions concerning extinction of magnon modes in the dynamical structure factor are observed using

the spin model with all the magnetic sublattices and the magnetic interactions derived from DFT calculations.

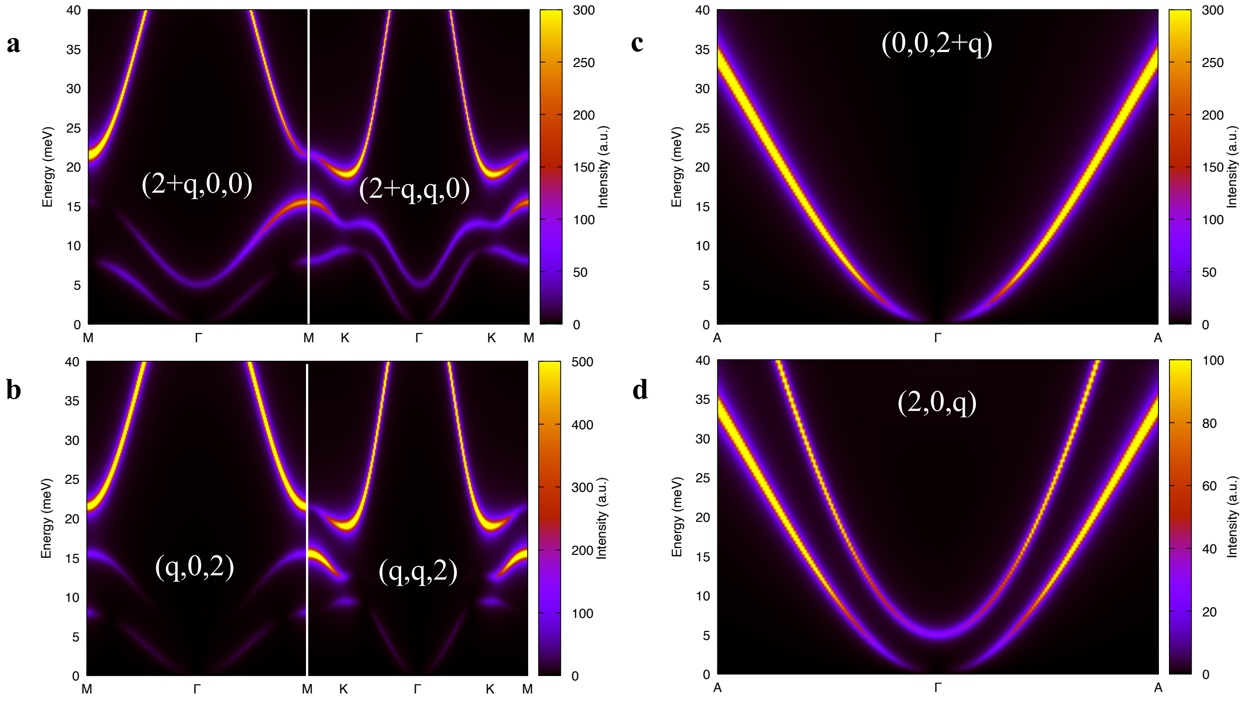

Fig. S15. **Simulated INS using the simplified spin model. a-d** Show the simulated dynamical structure factor in different Brillouin zones, following Eq. (S44), and neglecting the atomic magnetic form factor. The model is described in Section II E.

Within the presented theoretical approach, the dynamical structure factor is given by Eq. (S44). Mathematically, the quantity responsible for constructive/destructive interference is the factor $e^{\mathbf{ik}\cdot(\mathbf{R}_\mu-\mathbf{R}_\nu)}$ which arises from the incoming and diffracted waves, summed over the different magnetic sublattices μ, ν. This also makes the results dependent on the chosen Brillouin zone (centered around a given reciprocal lattice vector), while the intrinsic dynamical spin susceptibility is independent of this choice.

INS measurements have been carried out around $(2+q,0,0)$ (see Figs. S7), $(2+q,q,0)$ (see Figs. S8), $(0,0,2+q)$ (see Figs. S4), $(q,0,2)$ (see Figs. S6(a),(c)) and $(q,0,3)$ (see Fig. S6(b)). We show the corresponding simulated INS in Fig. S15, together with some unmeasured directions. For $(2+q,0,0)$ and $(q,0,2)$, Fig. S15(a,b), three modes are visible near the M-point, as in the experiment (see Fig. S6(c) and Fig. S7(b)). For $(2+q,q,0)$ three modes can be observed (see Fig. S8(b)), although the acoustic mode is not seen to

approach Γ with observable intensity, in contrast to the INS data (see Fig. S8(a)). As found experimentally (see Fig. S6(b)), no magnon excitations are observable for $(q, 0, 3)$ (simulated INS not shown). Fig. S15(c) confirms that only one magnon band is observed for $(0, 0, 2+q)$ (the $\Gamma - A$ path in Fig. 2(b) of the main text and Figs. S4), while both branches should be visible for $(2, 0, q)$ as shown in Fig. S15(d).

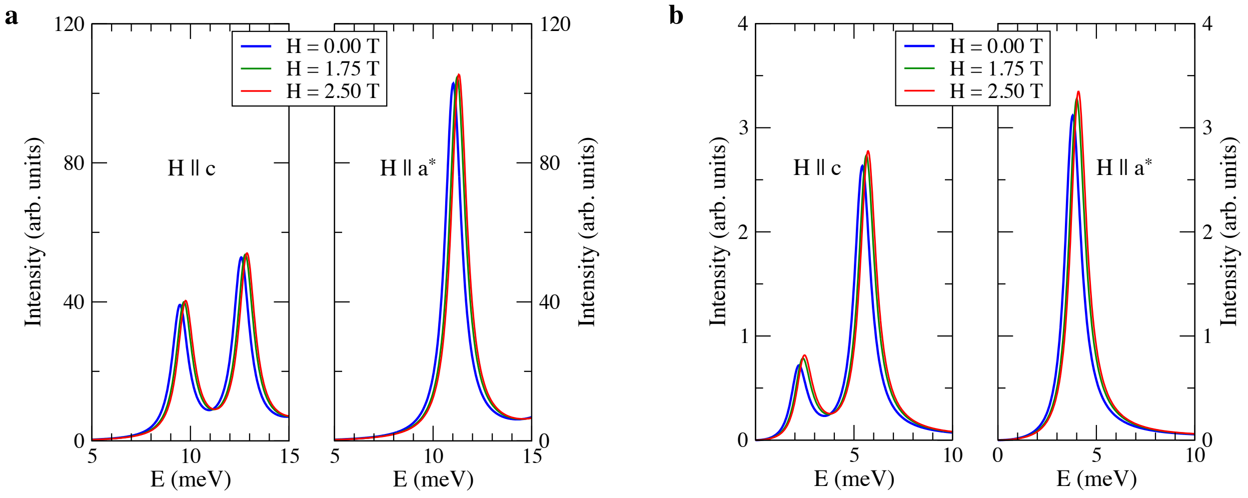

Fig. S16. **Simulated constant Q-scans at the K-point Q** $= (2.333, 0.333, 0)$. **a** Using the simplified spin model. **b** Using the magnetic interactions obtained from DFT with the experimental crystal structure.

We show in Figs. S16 simulated constant-**Q** cuts for $\mathbf{Q} = (2.333, 0.333, 0)$ and different orientations of the magnetization/applied field, similar to the experimental ones in Figs. 3(b,c) of the main text, using the simplified spin model and also the model parameterized with the interactions from DFT employing the experimental crystal structure. For $\mathbf{H} \parallel \mathbf{c}$, the intensities of two magnon modes are much more different in the case of the DFT model than in the case of the simplified model, which is due the different precessional motions of the respective magnons as obtained from each model. A direct comparison with the experimental data is not attempted, due to uncertainties in how to quantitatively model the broadening of the modes (which goes beyond the present scope of the theory), besides the limitations introduced by the assumptions inherent to the construction of the models themselves. However, in both cases we find that the gap between the two magnon modes indeed closes for $\mathbf{H} \parallel \mathbf{a}^*$, as predicted on general ground and found in our experimental measurements.

SUPPLEMENTARY REFERENCES

[1] N. Maraytta, J. Voigt, C. Salazar Mejía, K. Friese, Y. Skourski, J. Perßon, S. M. Salman, and T. Brückel, Anisotropy of the magnetocaloric effect: Example of Mn_5Ge_3, J. Appl. Phys. **128**, 103903 (2020).

[2] K. Schmalzl, W. Schmidt, S. Raymond, H. Feilbach, C. Mounier, B. Vettard, and T. Brückel, The upgrade of the cold neutron three-axis spectrometer IN12 at the ILL, Nucl. Instrum. Methods Phys. Res. A **819**, 89 (2016).

[3] E. J. Lisher and J. B. Forsyth, Analytic approximations to form factors, Acta Crystallographica Section A **27**, 545 (1971).

[4] S. Petit, F. Moussa, M. Hennion, S. Pailhès, L. Pinsard-Gaudart, and A. Ivanov, Spin Phonon Coupling in Hexagonal Multiferroic $YMnO_3$, Phys. Rev. Lett. **99**, 266604 (2007).

[5] A. S. Sukhanov, M. S. Pavlovskii, P. Bourges, H. C. Walker, K. Manna, C. Felser, and D. S. Inosov, Magnon-polaron excitations in the noncollinear antiferromagnet Mn_3Ge, Phys. Rev. B **99**, 214445 (2019).

[6] S. V. Dordevic, L. W. Kohlman, N. Stojilovic, R. Hu, and C. Petrovic, Signatures of electron-boson coupling in the half-metallic ferromagnet Mn_5Ge_3: Study of electron self-energy $\Sigma(\omega)$ obtained from infrared spectroscopy, Phys. Rev. B **80**, 115114 (2009).

[7] W. Ndiaye, M. C. Richter, O. Heckmann, P. De Padova, J.-M. Mariot, A. Stroppa, S. Picozzi, W. Wang, A. Taleb-Ibrahimi, P. Le Fèvre, F. Bertran, C. Cacho, M. Leandersson, T. Balasubramanian, and K. Hricovini, Bulk electronic structure of Mn_5Ge_3/Ge(111) films by angle-resolved photoemission spectroscopy, Phys. Rev. B **87**, 165137 (2013).

[8] C. Mangold, S. Chen, G. Barbalinardo, J. Behler, P. Pochet, K. Termentzidis, Y. Han, L. Chaput, D. Lacroix, and D. Donadio, Transferability of neural network potentials for varying stoichiometry: Phonons and thermal conductivity of Mn_xGe_y compounds, J Appl Phys **127**, 244901 (2020).

[9] G. L. Squires, *Introduction to the Theory of Thermal Neutron Scattering* (Cambridge University Press, 2012).

[10] S. Raymond, N. Biniskos, K. Schmalzl, J. Persson, and T. Brückel, Total interference between nuclear and magnetovibrational one-phonon scattering cross sections, J. Phys.: Conf. Ser. **1316**, 012018 (2019).

[11] J. A. Fernandez-Baca, R. M. Nicklow, Z. Tun, and J. J. Rhyne, Neutron-scattering study of the magnetic excitations of thulium metal, Phys. Rev. B **43**, 3188 (1991).

[12] O. Steinsvoll, R. M. Moon, W. C. Koehler, and C. G. Windsor, Magnetic form factor of metallic iron and nickel as seen by inelastic neutron scattering from phonons, Phys. Rev. B **24**, 4031 (1981).

[13] P. J. Brown, B. Roessli, J. G. Smith, K.-U. Neumann, and K. R. A. Ziebeck, Determination of the wavevector and temperature dependence of the 'forbidden' mode in $Fe_{65}Ni_{35}$ Invar using inelastic neutron scattering, J. Phys.: Condens. Matter **8**, 1527 (1996).

[14] P. Giannozzi, S. Baroni, N. Bonini, M. Calandra, R. Car, C. Cavazzoni, D. Ceresoli, G. L. Chiarotti, M. Cococcioni, I. Dabo, A. D. Corso, S. de Gironcoli, S. Fabris, G. Fratesi, R. Gebauer, U. Gerstmann, C. Gougoussis, A. Kokalj, M. Lazzeri, L. Martin-Samos, N. Marzari, F. Mauri, R. Mazzarello, S. Paolini, A. Pasquarello, L. Paulatto, C. Sbraccia, S. Scandolo, G. Sclauzero, A. P. Seitsonen, A. Smogunov, P. Umari, and R. M. Wentzcovitch, QUANTUM ESPRESSO: a modular and open-source software project for quantum simulations of materials, J. Phys.: Condens. Matter **21**, 395502 (2009).

[15] K. F. Garrity, J. W. Bennett, K. M. Rabe, and D. Vanderbilt, Pseudopotentials for high-throughput DFT calculations, Comput. Mater. Sci. **81**, 446 (2014).

[16] J. P. Perdew, K. Burke, and M. Ernzerhof, Generalized Gradient Approximation Made Simple, Phys. Rev. Lett. **77**, 3865 (1996).

[17] J. B. Forsyth and P. J. Brown, The spatial distribution of magnetisation density in Mn_5Ge_3, J. Phys.: Condens. Matter **2**, 2713 (1990).

[18] N. Papanikolaou, R. Zeller, and P. H. Dederichs, Conceptual improvements of the KKR method, J. Phys.: Condens. Matter **14**, 2799 (2002).

[19] D. S. G. Bauer, *Development of a relativistic full-potential first-principles multiple scattering Green function method applied to complex magnetic textures of nano structures at surfaces*, Ph.D. thesis, RWTH Aachen (2014).

[20] `https://jukkr.fz-juelich.de/`.

[21] S. Vosko, L. Wilk, and M. Nusair, Accurate spin-dependent electron liquid correlation energies for local spin density calculations: a critical analysis, Can. J. Phys. **58**, 1200 (1980).

[22] K. Wildberger, P. Lang, R. Zeller, and P. Dederichs, Fermi-dirac distribution in ab initio Green-function calculations, Phys Rev B **52**, 11502 (1995).

[23] H. Ebert and S. Mankovsky, Anisotropic exchange coupling in diluted magnetic semiconductors: Ab initio spin-density functional theory, Phys. Rev. B **79**, 045209 (2009).

[24] T. Moriya, Anisotropic Superexchange Interaction and Weak Ferromagnetism, Phys. Rev. **120**, 91 (1960).

[25] I. Slipukhina, E. Arras, P. Mavropoulos, and P. Pochet, Simulation of the enhanced Curie temperature in $Mn_5Ge_3C_x$ compounds, Appl Phys Lett **94**, 192505 (2009).

[26] F. J. dos Santos, M. dos Santos Dias, F. S. M. Guimarães, J. Bouaziz, and S. Lounis, Spin-resolved inelastic electron scattering by spin waves in noncollinear magnets, Phys. Rev. B **97**, 024431 (2018).

[27] N. Biniskos, S. Raymond, K. Schmalzl, A. Schneidewind, J. Voigt, R. Georgii, P. Hering, J. Persson, K. Friese, and T. Brückel, Spin dynamics of the magnetocaloric compound $MnFe_4Si_3$, Phys. Rev. B **96**, 104407 (2017).
